# Consensus Among International Ethical Guidelines for the Provision of Videoconferencing-Based Mental Health Treatments

**DOI:** 10.2196/mental.5481

**Published:** 2016-05-18

**Authors:** Ursula M Sansom-Daly, Claire E Wakefield, Brittany C McGill, Helen L Wilson, Pandora Patterson

**Affiliations:** ^1^ Discipline of Paediatrics, School of Women's and Children's Health UNSW Medicine University of New South Wales Randwick Australia; ^2^ Behavioural Sciences Unit Kids Cancer Centre Sydney Children's Hospital Randwick Australia; ^3^ Sydney Youth Cancer Service Sydney Children's/Prince of Wales Hospitals Randwick Australia; ^4^ CanTeen Australia Sydney Australia; ^5^ Cancer Nursing Research Unit Sydney Nursing School University of Sydney Sydney Australia

**Keywords:** Internet, videoconferencing, Skype, mental health, cognitive therapy, ethics, professional, guidelines as topic, professional practice, societies, standards

## Abstract

**Background:**

Online technologies may reduce barriers to evidence-based mental health care, yet they also create numerous ethical challenges. Recently, numerous professional organizations and expert groups have produced best-practice guidelines to assist mental health professionals in delivering online interventions in an ethically and clinically sound manner. However, there has been little critical examination of these international best-practice guidelines regarding appropriate electronic mental health (e-mental health) service delivery via technologies such as videoconferencing (including Skype), particularly for specific, vulnerable populations. Further, the extent to which concordance exists between these guidelines remains unclear. Synthesizing this literature to provide clear guidance to both mental health professionals and researchers is critical to ensure continued progress in the field of e-mental health.

**Objective:**

This study aims to review all currently available ethical and best-practice guidelines relating to videoconferencing-delivered mental health treatments in order to ascertain the recommendations for which international consensus could be found. Additionally, this review examines the extent to which each set of guidance addresses several key special populations, including children and young people, and populations living with illness.

**Methods:**

This systematic review examined guidelines using a two-armed search strategy, examining (1) professional organizations’ published guidance; and (2) MEDLINE, PsycINFO, and EMBASE for the past ten years. In order to determine consensus for best-practice, a recommendation was considered "firm" if 50% or more of the reviewed guidelines endorsed it and "tentative" if recommended by fewer guidelines than these. The professional guidelines were also scored by two raters using the Appraisal of Guidelines for Research and Evaluation II (AGREE-II) criteria.

**Results:**

In the study, 19 guidelines were included, yielding 11 specific "firm" and a further 123 "tentative-level" recommendations regarding the appropriateness of e-mental health, competence, legal and regulatory issues, confidentiality, consent, professional boundaries, and crisis management. International consensus yielded firm guidance across almost all areas except professional boundaries and some aspects of determining the appropriateness of e-mental health. Few guidelines specifically addressed special populations. Overall guideline quality varied; however, 42% (8/19) of the guidelines scored at least 5 out of 7.

**Conclusions:**

This synthesis of guidelines provides a foundation for clinicians and researchers utilizing e-mental health worldwide. The lack of specific guidance relating to special populations is an area warranting further attention in order to strengthen mental health professionals’ and researchers’ capacity to ethically and effectively tailor e-mental health interventions to these groups.

## Introduction

The increasing burden of mental health disorders has focused attention on the need for evidence-based psychological services [1]. Internationally, there remain numerous barriers to accessing evidence-based, specialist mental health interventions, including distance, financial access and/or cost, and stigma [2]. Electronic mental health (e-mental health) strategies have significant potential to address these issues through reducing or removing these barriers [3,4]. Building on previous definitions [5], e-mental health refers here to the provision of mental health services delivered or enhanced by Internet-related technologies; including email, chat, videoconferencing (for example, via Skype), and websites. E-mental health services may be synchronous with two or more parties interacting in real-time (eg, videoconferencing), asynchronous where there may be delays between two parties’ communications (eg, email), or involve no clinician interaction whatsoever (eg, self-guided modules). E-mental health can be used as an alternative to traditional face-to-face (FTF) support or as an adjunct to routine practice - for example, for follow-up sessions, or as initial treatment within a stepped care model [6]. In this way, e-mental health has significant potential to increase the accessibility of evidence-based interventions in increasingly cost-constrained health settings [7].

Early evidence across case studies, randomized controlled trials (RCTs), and meta-analyses point to e-mental health’s potentially equivalent therapeutic benefits to FTF formats [8-12]. Therapist-assisted models show the strongest effects [13,14]. Qualitative and quantitative evaluations of e-mental health’s acceptability, feasibility, safety, and process elements such as working alliance are also promising [8,15]. Both child and adolescent [16-19], and adult [1] clients have indicated that e-mental health methods are as satisfactory as FTF, and reduce the burden of travel.

Despite this burgeoning literature, many recent critical examinations argue that there is a lack of high-level, gold-standard evidence to support the efficacy of e-mental health. Most studies have compared e-mental health interventions to waitlist controls, rather than the gold-standard attention, in vivo control [9,20,21]. A recent review of videoconference-delivered treatment for anxiety disorders delivered in the last decade indicated that videoconference-delivered treatment is effective in reducing symptoms of anxiety, and that the outcomes are comparable with those found in FTF formats. However, this evidence does not exist for all mental health issues, and the general uncertainty regarding the management of ethical quandaries in e-mental health forms a significant barrier to generating further high-quality research [2,9], and may also add to clinicians’ hesitance to try this mode of delivering therapy. This has contributed to a lag in the uptake of these technologies, even in settings where they are readily available [3,22,23].

While a range of e-mental health modalities exist, each with unique clinical applications and ethical implications, videoconferencing has emerged as an especially promising modality with growing research and clinical use. Videoconferencing has already been employed for many years [10-12], in both practitioner-patient and supervision areas, and has a rapidly growing evidence base [24]. That videoconferencing most closely mirrors a traditional FTF session may also mean that it is the modality that professionals are most likely to integrate into their routine practice.

### Ethical Implications of E-Mental Health

As e-mental health interventions become increasingly advanced, researchers have begun to consider their vast ethical implications [2,9]. While the ethical issues relevant to FTF services still apply to e-mental health, there are unique issues to consider with this new modality. Because of the use of technology, there are additional legal (confidentiality, obtaining consent, licensing, and record keeping) and professional issues (privacy and maintaining professional boundaries) to address. Pertaining to the provision of services remotely, the issues to be considered include crisis risk management and appropriateness of the service for the client. The practitioner must also be competent in delivering the service via this modality [25]. More specifically, videoconferencing requires careful consideration of ethical issues relating to confidentiality (of the session, other correspondence, and patient notes), competence, and consent [25].

However, the rapid expansion of online interventions has somewhat outpaced regulatory bodies’ capacity to provide guidance around their provision [9,26,27]. Although several professional bodies have issued guidelines relevant to e-mental health, it remains unclear whether there is international consensus regarding how potential ethical issues should be managed in practice [9,21]. The capacity for online interventions to be accessed by consumers across state and country lines, in conjunction with possible jurisdictional differences, speaks to the need for the reconciliation of available guidelines [2,9]. Identifying consensus among ethical practice guidelines is critical for ensuring the efficacy of online interventions and for the protection of both client and psychologist [28]. The availability of e-mental health has the potential to increase the reach of psychological services to consumers, however, with this there are new ethical issues and risks. As with any area of mental health service, there may be great risk with the provision of services without proper ethical guidance. Perhaps this is even more pertinent in the online arena, where for example in telepsychology, there is an increased risk of confidentiality breaches [29]. It is critical that the ethical issues unique to this new modality of videoconferencing are discussed and addressed. Special consideration also needs to be given to how these ethical codes might apply within different settings and to special populations [9,30].

### Special Populations

Mental health professionals may require additional, tailored guidance regarding the provision of e-mental health services to populations regarded as high risk, including individuals with psychosis or suicidal tendencies [31]. Other special populations with unique considerations include individuals with co-morbid serious or chronic illness, as well as children and young people [32-35]. Specialized mental health support tailored to the needs of these groups may be difficult to access outside of metropolitan regions. Individuals with a chronic illness show an estimated 10% higher incidence of mental disorders relative to healthy individuals [36], and the burden of physical symptoms may make the accessibility afforded by e-mental health especially relevant to this group. Similarly, e-mental health may address the barriers of stigma and geographic isolation in young people accessing evidence-based interventions from mental health professionals [32,35,37], and may even promote further help-seeking [38,39].

### The Present Review

This review focuses on guidelines for delivering videoconferencing-based treatments (for example, via Skype), because this modality requires the active participation of a mental health professional in real-time, in the same way as a FTF session. Focusing on this modality allowed this review to generate more specific recommendations that were more easily applicable to practice. Discussions of the ethics of other forms of e-mental health are available elsewhere [40,41].

This systematic review aims to identify consensus among current best-practice guidelines and synthesize the recommendations regarding the ethical delivery of mental health treatments using videoconferencing technology. The review aims to answer the following two key research questions: (1) what areas of consensus in recommendations can be identified from international best-practice guidelines to guide clinicians in the ethical provision of videoconferencing-based mental health treatments? (2) What recommendations are made in best-practice guidelines regarding special populations including high risk, unwell, and young people?

## Methods

The review was conducted in accordance with the Preferred Reporting Items for Systematic Reviews and Meta-Analyses (PRISMA) guidelines for systematic reviews [42].

### Search Strategy

In order to integrate research evidence with best-practice guidelines and capture the broadest set of current guidance, a two-armed approach was adopted involving systematic searches of (1) guidelines disseminated by international mental health-related professional bodies; and (2) the recent peer-reviewed literature for best-practice guidelines and recommendations published in scientific journals.

#### Search One: Professional Bodies and Organizations

We searched the websites of peak professional bodies in countries where videoconferencing-based mental health interventions are known to be occurring; as informed by a recent review [8]. The websites of other international professional bodies affiliated with the American Psychological Association (APA) were also identified through their website [43] and subsequently searched. These organizations were individually searched for the presence of an English-language website, and the availability of ethical guidelines specific to e-mental health and videoconferencing-based interventions. Guidelines publicly available in draft format were also included where no more recent document existed.

#### Search Two: Published Expert Recommendations

**Figure 1:**
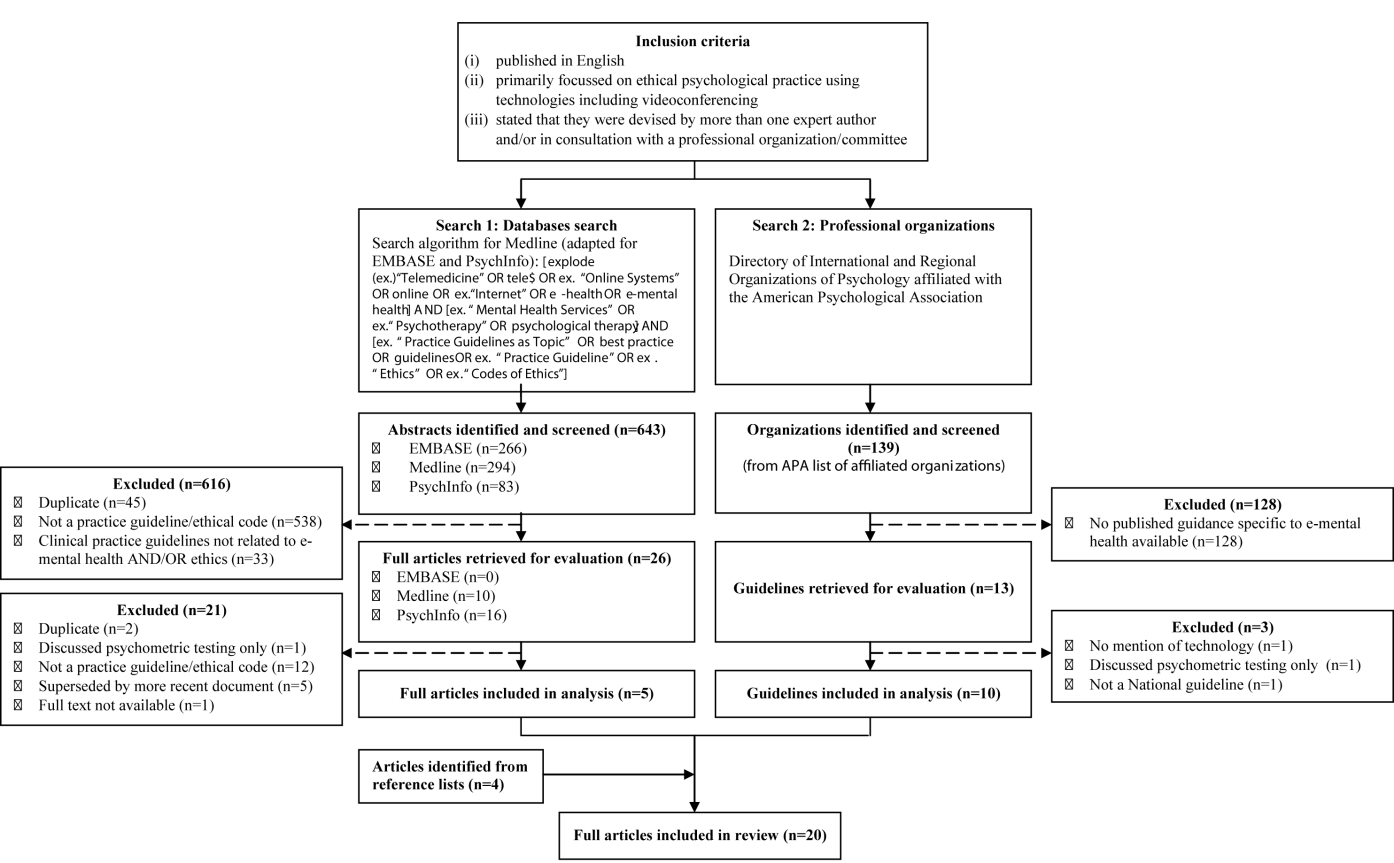
Preferred Reporting Items for Systematic Reviews and Meta-Analyses (PRISMA) flowchart of the search and selection process of guidelines included in the review.

We also undertook a targeted search of the peer-reviewed scientific literature for published recommendations generated by expert groups. Three electronic databases were searched (Medline, EMBASE, and PsychInfo), for human studies published in English between 2004 and 2014. The search and selection process, with detailed exclusion criteria is depicted in Figure 1. Search terms were informed by recent reviews [8,16], and included terms defining online interventions, psychology and mental health, and best-practice-recommendations or guidelines. We did not specify videoconferencing as a search term to avoid missing relevant papers in an emerging research field, where at present most guidelines are general to a variety of forms of e-mental health. Reference lists of reviews retrieved were also screened to identify additional relevant guidelines. An initial abstract review identified potentially eligible articles, in addition to a subsequent full-text review by two independent reviewers (ER and MB). Here, articles including best-practice guidance and clinical recommendations relevant to videoconferencing were included. Reviewer disagreements regarding article inclusion were discussed further to achieve consensus, remaining disagreements were resolved through consultation with the other authors.

### Inclusion Criteria

Best-practice guidelines were included if they (1) were published in English; (2) focused on ethical practice recommendations to deliver therapeutic mental health services using online technologies (including videoconferencing); and (3) stated that they were devised by more than one expert author and/or in consultation with a national professional organization and/or committee.

### Data Synthesis and Method for Establishing Consensus

The inter-rater reliability for the guideline search was acceptable (83%). The following data was extracted from guidelines: organization/lead author, year of publication and review, country of origin, guideline type, technological focus, target audience, and peer-reviewed status or involvement of consumer input (Multimedia Appendix 1). In order to be considered peer-reviewed, guidelines needed to explicitly state that they underwent a process during development whereby recommendations were reviewed by colleagues, other relevant organizations or stakeholders. Ethical recommendations from each guideline were extracted and collated by content (ie, ethical issue) by two authors (USD, HW). Each individual recommendation was read for specific intent and guidance, with recommendations with the same essential intent collapsed across guidelines (Multimedia Appendices 2-8). In seeking to establish consensus where possible, this review applied a graded rating scale across each recommendation. We considered recommendations made by 50% or more of the expert groups to be firm recommendations, the highest level of evidence-consensus (described as "was recommended"). As this is a new area of research, a consensus seen across half (50%) or more of the existing expert groups was considered representative of consistent recognition of a particular issue, particularly given the varying scope of the 19 international guidelines included. We categorized recommendations proposed by fewer groups than this as tentative ("may be recommended").

### Quality Assessment

The quality of guidelines published by professional organizations (identified through Search Two) was also assessed by two researchers (USD and HW) using the Appraisal of Guidelines for Research and Evaluation (AGREE-II) instrument [44]. The AGREE-II tool assesses the methodological rigor of guidelines using 23 items rated across six domains: scope and purpose, stakeholder involvement, rigor of development, clarity of presentation, applicability, and editorial independence (including bias). Guideline quality was not a criterion for review inclusion.

## Results

### Guidelines and Position Papers

The guideline selection and inclusion process are depicted in Figure 1. We included 19 different guidelines or position papers (Multimedia Appendix 1), originating from seven countries, with the majority from USA (53%, 10/19). All guidelines included information on videoconferencing-based mental health treatments. Most were targeted towards psychologists (74%, 14/19) (Multimedia Appendix 9). As online psychological therapy should be conducted by an appropriately trained, accredited mental health professional [27], this review does not include recommendations for para-psychology or other professionals (eg, teachers, nurses). The qualitative assessment did not reveal any issues of bias across studies.

### Quality Assessment

There was significant variation between guidelines in the breadth and depth of topics covered across guidelines (Multimedia Appendix 9). The AGREE-II [44] tool highlighted significant variation in the quality of the 14 guidelines produced by professional organizations, often due to guidelines failing to explicitly address certain elements. Mean overall quality scores ranged from 2-6 out of 7 (scale ranges from 1 "lowest possible quality" to 7 "highest possible quality"), with 64% (9/14) professional guidelines recommended for use on the basis of these scores [45-54]. The domain relating to guidelines’ *Scope and Purpose* showed the highest overall quality, with a mean score of 76.6% across guidelines (range 33-100%), while the domain of *Editorial Independence* appeared the least well addressed across guidelines (mean score 2%; range 0-33%), which could reflect the methods of review used by professional organizations in publishing their guidelines. As previously mentioned, the AGREE-II assessment was not used as criteria for inclusion or exclusion. In addition to the AGREE-II criteria, guidelines were also examined in terms of whether their development had involved peer-review and consumer involvement. Only 32% (6/19) guidelines explicitly described processes of seeking peer-review from expert colleagues as part of their development [27,28,48,51,53,55], while only one set of guidelines reported involving consumers as part of their expert committee (Multimedia Appendix 1) [51].

### Research Question One

#### What Recommendations can be Drawn From the Guidelines?

All of the guidelines reviewed indicated that mental health professionals overarching ethical obligations do not change when services are being delivered using online modalities. A summary of the ethical issues addressed across guidelines is shown in Multimedia Appendix 9. A detailed summary of specific firm recommendations for which consensus was found (across 10 or more of the 19 guidelines), as well as tentative recommendations is presented in Multimedia Appendices 2-8 , grouped by the following general ethical domains: the appropriateness of e-mental health, legal considerations, confidentiality, consent, professional boundaries, and crisis management. In total, 11 specific firm recommendations could be drawn from the reviewed guidelines, relating to areas such as determining the appropriateness of e-mental health, competence to deliver e-mental health interventions, legal and regulatory issues, confidentiality, consent, and crisis interventions and distress management. No firm recommendations could be drawn relating to professional boundaries. A further 123 tentative-level recommendations were drawn across all domains. An overview of the recommendations with key practice recommendations highlighted is shown in Multimedia Appendix 10.

#### Appropriateness of e-Mental Health

##### Client-Related Factors

Although several sets of guidelines identified particular groups or individuals for whom e-mental health may be contraindicated, they differed in terms of how strongly they advised against providing e-mental health interventions to such individuals. The most strongly endorsed recommendation indicated that mental health professionals should incorporate an assessment process to determine the appropriateness of e-mental health services for an individual client (58%, 11/19 guidelines) (see Multimedia Appendices 2-8). However, of these, only four sets of guidelines provided more concrete recommendations as to how professionals could undertake such an assessment [46,52,54,55].

##### Service-Related Factors

No firm recommendations emerged regarding the ethical implications of the psychological service type being offered (Multimedia Appendices 2-8). Several recommendations received tentative-level endorsement; however, the most strongly endorsed of these suggested that psychological tests designed to be implemented FTF may not be possible or ethical to conduct online. Only three sets of guidelines commented on the appropriateness of delivering other psychological services using e-mental health (eg, couples, family, or group-based therapy), however, few specific recommendations were generated [46,52,56].

#### Competence

Regarding general professional competence, three firm recommendations emerged. The two most strongly endorsed of these indicated that mental health professionals should provide online services within the boundaries of their competence, with an understanding of the limits and applications of different technologies (58%, 11/19 guidelines), and that they should also assist clients to readily assess their competence by verifying their identity and making available their qualifications and professional endorsements (58%, 11/19 guidelines) (Multimedia Appendix 3). Regarding technical competence, one firm recommendation emerged indicating that professionals should acquire skills to manage the technology they are using and to navigate core interpersonal, therapeutic processes using e-mental health services (53%, 10/19 guidelines). No firm recommendations could be drawn regarding competence to assess the suitability of an e-mental health service for a particular client or group. However, the strongest tentative-level recommendation indicated that mental health professionals should be culturally competent to deliver online services to different populations, including considerations of clients’ ethnic/racial, cultural, linguistic, gender/sexual orientation, geographic, and socioeconomic backgrounds (42%, 8/19 guidelines).

#### Legal and Regulatory Issues

Guidelines highlighted recommendations related to the psychologist’s accreditation, record keeping, billing, and establishing a client’s age to determine their (legal) capacity to consent (Multimedia Appendix 4). Two firm recommendations emerged relating to mental health professionals’ registration/accreditation; these indicated that professionals should know and comply with all relevant laws and regulations (from both their and their client’s jurisdiction; 79%, 15/19 guidelines), and should ensure that their licensing board approves the provision of online services, and obtain site-specific credentialing across jurisdictions where necessary (63%, 12/19 guidelines). No firm-level recommendations emerged regarding record keeping and electronic records; however, the strongest tentative-level recommendation highlighted that professionals delivering e-mental health interventions should adhere to the usual laws and professional standards applicable to record keeping, particularly where the intervention diverges from usual practice (47%, 9/19 guidelines). Regarding *billing*, no firm recommendations emerged; the strongest tentative-level recommendation highlighted that mental health professionals should clarify up-front the nature and security of payment for sessions (21%, 4/19 guidelines). Finally, in determining a client’s legal capacity to consent to e-mental health interventions, no firm recommendations emerged. However, the two strongest tentative-level recommendations highlighted that professionals should take steps to determine the age of potential clients to establish the appropriateness of e-mental health interventions, and should ensure that a parent/guardian’s consent is obtained for all minors before services proceed (both 21%, 4/19 guidelines).

#### Confidentiality

Guidelines examined several aspects of confidentiality, including privacy during the online session, client anonymity and establishing identity, and appropriate use and storage of electronic material (Multimedia Appendix 5). One firm recommendation emerged, indicating that mental health professionals should take all up-to-date precautionary efforts to protect clients’ confidentiality using e-mental health services (63%, 12/19 guidelines). Further tentative-level guidance emerged pertaining to the issues of ensuring privacy during e-mental health sessions, client anonymity and establishing identity, and confidentiality in the use and storage of electronic materials (Multimedia Appendix 5).

#### Consent

Guidelines regarding consent addressed the limits to confidentiality across technologies, the need to clarify expected psychologist-client contact, and a client’s capacity to legally consent (Multimedia Appendix 6). Two firm recommendations emerged regarding *limits to confidentiality*, highlighting the importance of undertaking and documenting thorough consent processes consistent with relevant laws and regulations (58%, 11/19 guidelines). A second recommendation further suggested that these consent processes should address numerous issues unique to e-mental health services including privacy and confidentiality in the online domain, security steps taken, technological equipment and skills requirements, limits to communication, and reliability of the connection (63%, 12/19 guidelines). No firm recommendations emerged regarding *clarifying contact times*, though the two strongest tentative-level recommendations indicated that professionals should clarify contact information, as well as the nature of and expectations around therapeutic contact at the commencement of e-mental health interventions (47%, 9/19 guidelines), and that professionals should additionally clarify expected timeframes for their client receiving a response from them, as well as processes around emergency contacts (47%, 9/19 guidelines). No firm recommendations emerged regarding *capacity to provide consent*.

#### Professional Boundaries

Recommendations regarding professional boundaries emerged in two key areas: preventing boundary crossings, and social media, though no firm recommendations emerged (Multimedia Appendix 7). The two strongest tentative-level recommendations indicated that mental-health professionals consider the increased potential for boundary issues to arise using e-mental health (21%, 4/19 guidelines) and use the same level of professional language across all media as they would in person (21%, 4/19 guidelines).

#### Crisis Intervention and Distress Management

Recommendations surrounding the assessment and management of acute distress and mental health crises were addressed across most guidelines (79%, 15/19 guidelines) (Multimedia Appendix 9). The only firm recommendation related to crisis management strategies, and highlighted that professionals should establish in-person clinical supports in the client’s geographic location prior to initiating e-mental health services, in case of emergency (53%, 10/19 guidelines) (Multimedia Appendix 8). Tentative-level guidance was also available relating to the communication of crisis management strategies, and mental health professionals’ responsibilities regarding crisis management in the context of e-mental health. The strongest tentative-level guidance in these areas indicated respectively that professionals inform clients of alternative means of communication should the technology fail (42%, 8/19 guidelines), and that professionals be familiar with mandatory reporting and involuntary hospitalization laws both for their, and their client’s jurisdictions (21%, 4/19 guidelines).

### Research Question Two : What Recommendations Were Made Regarding Special Populations?

Few guidelines discussed applications to particular special populations. Only six discussed providing e-mental health services to clients with high risk features such as psychosis. Populations noted as being higher risk across guidelines included clients with cognitive impairments (21%, 4/19 guidelines) and psychotic disorders (16%, 3/19 guidelines). Although several guidelines indicated that it may be preferable to exclude these individuals from e-mental health services (Multimedia Appendices 2-8), one set of guidelines noted that there is no concrete evidence indicating which populations may benefit most, or may be harmed by, psychological therapy delivered via videoconferencing [54]. One set of guidelines mentioned conducting e-mental health with illness populations [46], but no recommendations were made regarding adaptations to suit these groups.

A minority of guidelines (32%, 6/19 guidelines) discussed the appropriateness of e-mental health services for young people, with only two explicitly noting its potential acceptability and capacity to increase access to appropriate services [48,52]. However, more guidance was available regarding the importance of ensuring capacity to consent with young people (42%, 8/19 guidelines) (Multimedia Appendix 9), as well as useful strategies to obtain consent when sessions occur online. Given that minors may be highly computer literate (and may present as adult), professionals were advised to explicitly check young people’s age and the required consent prior to services proceeding. All guidelines noted that the requirement for parent/guardian legal consent remained in e-mental health interventions (Multimedia Appendix 6), and one set of guidelines noted that in some circumstances it may be important to engage with and support a young person, even if this consent is not possible [52]. In this case, the guidelines emphasized that identifying real-world support services around the young person would be a key element to therapeutic intervention, as well as discussion and thorough documentation around risk management [52]. Guidelines did not address tailored strategies for young people with regards to managing the following issues: anonymity and verifying identity, safety and risk management, or maintaining professional boundaries.

## Discussion

This review examined and synthesized available guidelines to guide mental health professionals in ethically delivering e-mental health services, with a particular focus on videoconferencing. A total of 19 guidelines were reviewed from seven countries/regions. Across guidelines, consensus supported 11 firm ethical practice recommendations. To our knowledge, this is the first time ethical and best-practice guidelines regarding e-mental health have been synthesized on an international scale. The resulting firm recommendations indicate that despite numerous, distinct best-practice documents, there is emerging agreement across jurisdictions.

Despite this, no firm guidance could be drawn in several key areas, including how professionals should assess the suitability of e-mental health services to their client (Multimedia Appendices 2-8), record-keeping, electronic records and billing (Multimedia Appendix 3), client anonymity, establishing identity and ensuring confidentiality in the use and storage of electronic materials (Multimedia Appendix 5), clarifying contact times and a client’s capacity to consent (Multimedia Appendix 6), how professionals should negotiate preventing boundary crossings and social media (Multimedia Appendix 7), and a professional’s responsibilities with relation to crisis management strategies in e-mental health interventions (Multimedia Appendix 8). This lack of specific and consistent guidance could be due to the aforementioned outpacing of the uptake of telepsychology with the establishment of professional guidelines for use, and with it a smaller pool of experience to draw from in producing these guidelines. In addition, the lack of uptake by some professionals due to the absence of clear guidance on how to approach the ethical issues unique to telepsychology may also be contributing to the lag in the production of guidelines. Achieving consensus in these areas will be critical to providing professionals with the confidence to deliver services online and may also contribute to providing a robust practice framework against which more rigorous research in this area may be undertaken.

Discordance between recommendations was rare; although in some instances guidelines differed in how strongly they discouraged certain practices. The delivery of e-mental health services to high risk populations was one such area. Although there was some (tentative-level) guidance regarding how identifiable electronic data relating to client material should be managed, there was less consensus around the appropriateness of mental health professionals recording material from their sessions (eg, audio-visual recordings of a videoconferencing session) [57]. More rigorous guidance is needed given that there may be new benefits (eg, using recordings in professional development, supervision, or as medico-legal documentation) as well as risks (eg, confidentiality) [27].

Regulatory issues in providing e-mental health to clients in different jurisdictions pose ongoing challenges for the field. The firm recommendations that mental health professionals be appropriately registered across their own and their client’s jurisdiction may be difficult to enforce in practice [52]. One set of guidelines noted that "If you enter the state or country via technology, you may be deemed to be practicing in that country" [52], and indicated that complaints made against a clinician would likely be heard in the client’s jurisdiction. Some work appears to have been undertaken for North American psychologists, with the development of a chart summarizing temporary and guest licensure across 50 states [58]. The future resolution of such issues internationally is likely to be critical to professionals’ confidence in delivering these services.

### Special Populations

#### "High Risk" Populations

Of the few guidelines that directly addressed special populations some discordance was evident as to whether this constituted a contraindication. One group noted that currently there is no convincing evidence identifying any populations for whom e-mental health services would be especially harmful, and cited emerging evidence that in some circumstances e-mental health could be successfully implemented with psychotic patients [54]. Evidence is mixed regarding whether or not high risk groups should be excluded or whether in some cases there is merit in including them with appropriate precautions [31,59].

The issue of crisis and risk management was comprehensively addressed across 15 guidelines (Multimedia Appendix 9), and offered numerous concrete strategies professionals may use to continually monitor and ensure the safety of their client. Based on these guidelines, thorough assessment, collaboration with local services, and partnership with the client’s support person may be integral to the management and safety of clients using e-mental health. Useful data may come from emerging e-mental health programs targeted at individuals with suicidal thoughts [60].

#### Illness Populations

Only one guideline highlighted the applicability of e-mental health strategies to illness populations, but no guidelines addressed how (if at all) the proposed recommendations should be tailored to these groups’ needs. Despite this, the convenience of e-mental health may be especially helpful in removing barriers to care for clients experiencing significant physical symptoms, such as fatigue or pain, which may hinder access to a FTF session [32,34]. E-mental health may facilitate more specialized support tailored to illness-specific issues [2], and connect otherwise dispersed individuals living with the same illness. This in turn could address the documented unmet need to access peer-support (eg, in cancer patients) [61-63].

Though firm recommendations regarding illness populations could not be drawn from this review, emerging literature indicates the acceptability, feasibility, and potential efficacy of videoconferencing-based psychological support among individuals with illness [64,65]. Despite the added complexities involved in delivering psychological support to multiple distressed unwell individuals concurrently, we have recently developed an intervention with adolescents and young adults with cancer, and documented rigorous protocols to successfully navigate challenging ethical issues surrounding consent, confidentiality, and crisis management [33,66]. Further work in this area will strengthen the evidence base for these unique groups.

#### Children and Young People

Online psychological therapy programs for children and young people involve the same core components as for adults [67], and are gaining evidence [8,16]. However, there is a gap in understanding how ethical and best-practice recommendations in the online space apply to young people [9]. Determining capacity to consent, and arranging for appropriate parental consent if needed, are important issues to consider. Some guidelines propose incorporating a FTF contracting session prior to the commencement of any online services [2,50-52]; this would enable the psychologist (or a proxy) to ascertain the age of the young person and obtain informed consent.

In cases where the referral or presenting problem itself makes parental or guardian consent difficult or inappropriate (eg, physical or sexual abuse involving their parent or guardian), an initial risk assessment should involve consideration of the young person’s vulnerability, and access to any other forms of appropriate evidence-based supports in the absence of online services [52]. Choosing to leave a vulnerable young person without any appropriate FTF supports due to online services being deemed too risky without parental consent does not avoid any ethical risks, but rather appears to make a choice as to which risk is preferable.

An issue not addressed by the reviewed guidelines is that young people may have limited access to e-mental health platforms due to their limited autonomy, particularly around finances. For example, some young people may not have independent access to the Internet or to computers in private locations to enable confidential conversations.

Although guidelines tentatively suggested that routine practice should involve clients being identifiable (Multimedia Appendix 5), several groups noted that maintaining anonymity may be reasonable under some circumstances. A desire for anonymity may lead an individual to provide a pseudonym, obscure their face, or to not use a Web camera at all. This issue may be particularly relevant to young people who report being especially concerned about the stigma related to consulting with mental health professionals [37]. Several existing community-based e-mental health platforms provide anonymous psychological services to adolescents and young adults (eg, Headspace [68] and CanTeen [69] in Australia; Blah Therapy [70] and Teen Line [71] in the United States).

In adapting e-mental health guidelines to children and young people, maintaining appropriate professional boundaries appears crucial. Due to the developing nature of their cognitive, emotional and social competencies, and their lack of exposure to mental-health services, young people may not necessarily understand the concept of professional boundaries. The reviewed guidelines tentatively indicated ways in which professionals can maintain boundaries, including monitoring what information is available about them online, maintaining professional language across all media (eg, in short message service [SMS] text messages), and avoiding interactions on social media.

### Gaps in Translation of Guidance to Practice

Despite predictions that online methods will soon form an integral part of mainstream psychological practice [30], gaps remain between ethical best-practice and the development of the competencies mental health professionals require to follow these guidelines [72]. For example, consensus indicated that professionals must practice within their area of competence, and need to learn the unique skills involved in delivering interventions online. One recent Canadian guideline proposed competency standards for professionals delivering e-mental health [72], however, little other guidance exists. This is a critical issue in need of resolution [21,72]. Recognized standards as to what would constitute adequate preparatory training do not yet exist [53]. These gaps are likely to need to be bridged through professional development integrally related to each practice setting at a local, jurisdictional level [9,21,72]. Professional development would need to incorporate both how practice may be modified to suit unique aspects of videoconferencing, and how clinicians can balance competing ethical demands in this space [9,22,28].

Another critical issue in translating videoconferencing-based methods into practice is ensuring increased exposure to the medium. Even when mental health professionals gain familiarity with videoconferencing and when it is shown to result in equivalent outcomes, higher client satisfaction and lower costs, professionals still report preferring FTF methods [73]. Lack of practice-based exposure has been identified as a contributing factor here [22]. Several specific practice issues that have been noted within videoconferencing, such as additional anxiety on the psychologist’s part, lack of feedback, and increased demands to ensure client engagement [22,74]; training addressing these issues may increase professionals’ skills, and lower their resistance to incorporating videoconferencing-based methods into routine practice. Previous reviews have noted mental health professionals’ concerns that videoconferencing methods might phase out FTF treatments [1,22]. However, one of the greatest benefits to professionals will likely be its capacity to supplement FTF - for example as an adjunct to FTF sessions [6,75].

There are currently few training opportunities in e-mental health methods [76], yet standardized specialized graduate training programs will become crucial to the field as it continues to become part of standard psychological care [77,78]. One recent Australian example has been reported, where videoconferencing-based training clinics were built into tailored postgraduate clinical psychology programs [79]. Videoconferencing has also been used to disseminate evidence-based professional development for mental health professionals [80]. The development of similar opportunities for practice and supervision in e-mental health methods is an important area for future development [2].

### Strengths and Limitations

This review represents the first time best-practice recommendations for the delivery of e-mental health interventions using videoconferencing have been evaluated for consensus across internationally-available literature. The review’s two-armed methodology enabled a synthesis of available guidance generated both by professional bodies as well as expert groups’ published recommendations. This was critical to ensure that consensus was drawn across the broadest set of currently-available guidance; this also increases the applicability of this review to future clinical practice and research endeavors in e-mental health.

Nevertheless, some limitations must be acknowledged. Guidelines currently in development may have been missed, and guidelines not published in English were excluded. The need to draw consensus across guidelines precluded the inclusion of great detail here, which may be useful to the clinician wishing to implement ethical guidance in their practice (though see Multimedia Appendices 2-8). In addition, guideline quality was only assessed for best-practice guidelines published by professional organizations, in keeping with the AGREE-II framework [44]. Nonetheless, professional guidelines varied significantly in their quality as well as their coverage of topics, an issue common to other guideline reviews [81].

Further, this review drew from a broader literature that is acknowledged to be in its infancy: a literature that suffers from several limitations itself. The ethical guidelines that do exist are not prescriptive but rather recommendations [21], with considerable gaps in how these recommendations might be adopted in varying contexts and populations [9]. In addition, one set of guidelines noted that while many ethical best-practice documents are somewhat abstract in nature, they require clinical, practical, or administrative guidance to be attached to them in order to be clinically useful [51].

For example, while many guidelines emphasized the importance of professionals continuously evaluating their competencies in delivering e-mental health interventions, including seeking ongoing training, few specific recommendations were made clarifying what precise interpersonal, therapeutic, or technical skills might be uniquely relevant to a clinician’s capacity to competently deliver e-mental health, or how this should be evaluated (either by professionals themselves or an external evaluator). This has implications for the future training of professionals in this field. National professional organizations’ capacity to create practice-based guidance for clinicians, as well as their capacity to develop and integrate practical materials into postgraduate training courses and accreditation and/or registration examinations as relevant, may be critical. As the literature base in this area develops, so too will clarity around what constitutes ethical best-practice for mental health professionals.

### Future Research

This exciting area of development offers numerous fruitful areas for future research. There is scope for international bodies to jointly participate in a Delphi consensus process [82] regarding best-practice e-mental health. Further work is needed to concretely determine the efficacy of videoconferencing-based services in larger populations, different disorders (including those classed as high risk), minority groups, using different therapy formats (eg, group-based), and compared to attention control groups. Future research should also consider more rigorously reporting on navigating consent in practice, as well as documenting clinical issues and adverse events that arise in the delivery of online services [27]. More detailed reports on what practical and technical logistics are required for implementation of these technologies are also needed, as many studies still do not report training, costs, and use of equipment within their interventions [8].

Online therapies have been heralded as a way to overcome perceived stigma around mental health [1,37]. Yet clients may be less likely to enter into a professional relationship with a psychologist if they have not had the opportunity to meet them FTF and evaluate their suitability for themselves [51]. Future research could explore whether e-mental health models show different rates of uptake and/or attrition relative to FTF therapy [1,37,51,83]. Some evidence also indicates that there is a higher rate of clients not returning for their second session in e-mental health [83]. Questions surrounding the ability to establish the therapeutic alliance as well as the quality and importance of this alliance in e-mental health interventions remain unanswered [27]; the cost-effectiveness of e-mental health services also needs to be established. A final question is whether e-mental health methods do in fact increase access to evidence-based services among vulnerable clients who are socio-economically or geographically disadvantaged, younger, or unwell.

### Conclusion

This review aimed to determine the consensus among international ethical guidelines regarding the provision of videoconferencing-based mental health services. Though different regulatory bodies across jurisdictions have unique local parameters within which professionals need to practice, consensus emerged across current international guidelines to inform how mental health professionals can effectively and ethically deliver their services using e-mental health platforms. Innovations in delivering mental health services online involve numerous trade-offs and the balance of different ethical risks, but if managed carefully may have significant benefits to a variety of vulnerable client groups.

## Appendix

Multimedia Appendix 1 Characteristics of the reviewed ethical guidelines and best-practice recommendations.

Multimedia Appendix 2 Specific recommendations for which consensus emerged regarding appropriateness of e-mental health.

Multimedia Appendix 3 Specific recommendations for which consensus emerged regarding competence.

Multimedia Appendix 4 Specific recommendations for which consensus emerged regarding legal and regulatory issues.

Multimedia Appendix 5 Specific recommendations for which consensus emerged regarding confidentiality.

Multimedia Appendix 6 Specific recommendations for which consensus emerged regarding consent.

Multimedia Appendix 7 Specific recommendations for which consensus emerged regarding professional boundaries.

Multimedia Appendix 8 Specific recommendations for which consensus emerged regarding crisis intervention and distress management.

Multimedia Appendix 9 Overview of ethical practice issues addressed across guidelines.

Multimedia Appendix 10 Most highly-endorsed recommendations across each ethical issue.

## Abbreviations

AGREE-II: Appraisal of Guidelines for Research and Evaluation II
e-mental health: electronic mental health
FTF: face-to-face
