# Supplementary material for: Consensus Among International Ethical Guidelines for the Provision of Videoconferencing-Based Mental Health Treatments
Source: JMIR Ment Health. 2016 May 18;3(2):e17. doi: 10.2196/mental.5481 (PMC4889868; doi:10.2196/mental.5481)
Supplement: Multimedia Appendix 1 [file mental_v3i2e17_app1.pdf]

Organization or lead author	Year	Last revised	Expiry/ next review date	Country	Guideline type	Peer-reviewed	Technological focus/scope	Target audience?	Consumer input?
American Counseling Association (ACA)	2014	2014	N/A	USA	Code of ethics – section on technolog-y	No*	Distance counselling, technology & social media	Counselors	No*
Association of Canadian Psychology Regulatory Organizations (ACPRO)	2011	N/A	N/A	Canada	Model standards	No	Telepsychology via "communicat-on technology"	Psychologi-sts	No
American Mental Health Counselors Association (AMHCA)	2010	2010	N/A	USA	Code of ethics – section on technolog-y	No	Technology-assisted counseling including computer, telephone, internet and other communication devices	Mental health counselors	No
American Psychological Association Joint Taskforce (APA-JT) for the Development of Telepsycholog y Guidelines for Psychologists	2013	N/A	N/A	USA	Practice recomme-ndations in peer-reviewed literature	Yes	Telepsychology : telephone, mobile devices, videoconferenci ng, e-mail, chat, text, internet (e.g. self-help, websites, blogs, social media)	Psychologi-sts	No
American Psychological Association (APA) Division 29	2011	N/A	N/A	USA	General practice recomme-ndations	No	Real-time sessions via phone and video	Psychologi-sts	No
Australian Psychological Society (APS)	2011	N/A	N/A	Australia	Practice recomme-ndations	No	Internet, e-mail, text messaging, telephone, skype, video conferencing	Psychologi-sts	No
American Telemedicine Association (ATA)	2013	N/A	N/A	USA	Practice recomme-ndations	No	Real-time internet-based videoconferenci ng, using computers and mobile devices	Health practitioners	No

**Organization or lead author**	**Year**	**Last revised**	**Expiry/ next review date**	**Country**	**Guideline type**	**Peer-reviewed**	**Technological focus/scope**	**Target audience?**	**Consumer input?**
American Telemedicine Association – South Africa (ATA-SA)	2011	N/A	N/A	South Africa	Practice guidelines	Yes	Videoconferen-cing, not delivered over Internet	Psychiatrists, mental health practitioners	No
The British Psychological Society (BPS)	2009	N/A	N/A	United Kingdom	Ethical practice recomme-ndations	No	Real-time internet-based videoconferenc e-ing (using computers and/or mobile devices), e-mail, websites, telephone	Psychologi-sts	No
Canadian Psychological Association (CPA)	2006	N/A	Draft version will be finalised with next issue of *Canadian Code of Ethics for Psychol-ogists*	Canada	Ethical practice recomme-ndations	No	Electronic media (e.g. telephone, videoconferenc e-ing, e-mail, chat rooms)	Psychologi-sts	No
European Federation of Psychologists' Association (EFPA)	2006	N/A	N/A	Europe	Ethical practice recomme-ndations	No	Internet (e.g. real-time software, e-mail), telephone, fax, TV, radio and written communication	Psychologi-sts	No
Joint International Society for Mental Health Online/ Psychiatric Society for Informatics (ISMHO/PSI)	2001	N/A	N/A	USA	Ethical practice recomme-ndations – peer reviewed literature	Yes	Online services (e.g. e-mail, videoconferen-cing)	Psychologi-sts/ Clinicians	Yes (ISMHO includes lay persons)

Organization or lead author	Year	Last revised	Expiry/ next review date	Country	Guideline type	Peer-reviewed	Technological focus/scope	Target audience?	Consumer input?
National Board for Certified Counselors (NBCC)	2001	N/A	N/A	USA	Standards of practice	No	Internet counselling, including chat, email- or video-based counselling for individuals, couples or groups	Primarily counsellors, but also clients, the public, educators, and relevant organization-ns delivering Internet counselling	No
New Zealand Psychologists Board (NZPB)	2011	N/A	Draft version to be finalised after Board's February 2012 meeting	New Zealand	Best practice recomme-ndations	No	Telepsychology: telephone, e-mail, videoconferenc-ing, texting, chat rooms, online message boards	Psychologi-sts	No
Dever Fitzgerald	2010	2010	N/A	Canada, USA	Ethical and legal considera-tions with practice recomme-ndations	No	Internet-based psychotherapy (IBP), involving web pages and self-help materials with varying degrees of therapist contact via e-mail, videoconferencing, and/or telephone	Psychologi-sts	No
Drum	2014	N/A	N/A	USA	Best-practice recomme-ndations – in peer-reviewed literature	Yes	Telepsychology: telephone, e-mail, text, videoconferenc-ing, mobile applications, and Web-based programs	Psychologi-sts	No
Lawlor-Savage	2014	2013	N/A	Canada	Ethical practice recomme-ndations – peer-reviewed literature	Yes	Digital cognitive behavioural therapy (CBT; computerised and/or internet-based)	Psychologi-sts	No

Organization or lead author	Year	Last revised	Expiry/ next review date	Country	Guideline type	Peer-reviewed	Technological focus/scope	Target audience?	Consumer input?
Johnson	2014	2014	N/A	Canada	Best practice recomme-ndations – peer-reviewed literature	Yes	Telepsychology: telephone, e-mail, chat, videoconferencing	Psychologi-sts	No
Luxton	2012	N/A	N/A	USA	Safety planning recomme-ndations	No	Synchronous, two-way home-based ‘telemental health’	Psychologi-sts, mental health clinicians	No

* Unless guidelines specifically noted having been peer-reviewed, or including consumer input, they were assumed not to have. ** In order to be considered ‘peer-reviewed’ here it was not sufficient for guidelines to be published in a peer-reviewed journal; rather, guidelines needed to explicitly state that they underwent a process during guideline development whereby their recommendations were reviewed by colleagues or other relevant organisations or stakeholders. N/A = Not available; USA = United States of America.
