# Supplementary material for: Consensus Among International Ethical Guidelines for the Provision of Videoconferencing-Based Mental Health Treatments
Source: JMIR Ment Health. 2016 May 18;3(2):e17. doi: 10.2196/mental.5481 (PMC4889868; doi:10.2196/mental.5481)
Supplement: Multimedia Appendix 2 [file mental_v3i2e17_app2.pdf]

	Professional bodies														Published recommendations				
**Recommendation**	**ACA**	**ACPRO**	**AMHCA**	**APA**	**APA D.29**	**APS**	**ATA**	**ATA -SA**	**BPS**	**CPA**	**EFPA**	**ISMHO & PSI**	**NBCC**	**NZPB**	**Dever**	**Drum**	**Johnson**	**Lawlor -Savage**	**Luxton**
**Ethical obligations do not change**		X	X	X		X	X	X	X		X		X	X					
Factors related to the client																			
**Firm recommendations**																			
Psychologists should incorporate an initial process of considering the appropriateness of online psychological therapy with an individual client and refer clients for whom online methods were not deemed to be suitable to alternative in-person support services	X		X	X		X	X	X						X	X		X	X	X
**Tentative recommendations**																			
Psychologists may consider whether online provision of their service is supported by research, or is even ‘contra-indicated’ for a particular disorder or psychological problem	X					X	X	X						X					X
Online therapeutic work may not be recommended with clients considered to be ‘high risk’					X		X							X					
Issues such as language/comprehension skills, cognitive issues/impairments, psychotic conditions including poor reality testing, borderline personality disorder, thought disorder, a history of violence, self-injurious behaviour or suicidal ideation, drug/alcohol issues, or unmonitored medical conditions, may be considered as factors that may contra-indicate online therapy	X						X							X					
Psychologists may also consider a patient’s expectations, and level of comfort with receiving psychological therapy ‘in the home’ in determining the appropriateness of videoconferencing, particularly given that this may increase the demands on the patient (organizationally and cognitively) as well as the consideration of establishing a private space in which their concerns may be discussed confidentially	X		X				X							X			X		X
The patient’s capacity to continue to arrange these practical and technological necessities, their technological skills, their geographic distance to their nearest emergency medical facility, as well as their capacity to continue to actively engage in managing their safety may be critical considerations to determining their candidacy for this type of therapy	X						X							X			X	X	X
It may be reasonable to recommend that psychologists continue to monitor and evaluate the above considerations throughout the course of the online therapy, possibly through the arrangement of a formal ‘trial period’							X							X					X
Psychologists may wish to have a pre-existing therapeutic relationship with a client prior to engaging in e-mental health methods if and when this is possible; however, for some clients with a disability, or located in a remote geographic location, online methods would be preferable and may preclude having a pre-existing ‘in person’ relationship					X														
Type of service																			
**Tentative recommendations**																			
It may be reasonable to recommend that psychologists be aware that psychological testing/assessments that are designed to be implemented in-person may not be possible or ethical to conduct online				X		X		X		X				X					
Psychologists may consider clearly communicate the inherent risks associated with online and unsupervised test administration to clients prior to administration						X								X					

	Professional bodies														Published recommendations				
**Recommendation**	**ACA**	**ACPRO**	**AMHCA**	**APA**	**APA D.29**	**APS**	**ATA**	**ATA -SA**	**BPS**	**CPA**	**EFPA**	**ISMHO & PSI**	**NBCC**	**NZPB**	**Dever**	**Drum**	**Johnson**	**Lawlor -Savage**	**Luxton**
Psychologists may consider ensuring that their client fits the target population that a test was designed for						X													

Note. The abbreviations in the table refer to the following articles. Please see article for full reference list.

ACA – American Counseling Association (ACA, 2014)

ACPRO – Association of Canadian Psychology Regulatory Organizations (ACPRO, 2011)

AMHCA – American Mental Health Counselors Association (AMHCA, 2010)

APA – American Psychological Association (APA, 2013)

APA D.29 - American Psychological Association Division 29 (APA, 2011)

APS – Australian Psychological Society (APS, 2011)

ATA – American Telemedicine Association (ATA, 2013)

ATA-SA – American Telemedicine Association – South Africa (Chipps, Ramlall & Mars, 2012)

BPS – The British Psychological Society (BPS, 2009)

CPA – Canadian Psychological Association (CPA, 2006)

EFPA – European Federation of Psychologists' Association (EFPA, 2006)

ISMHO/PSI – International Society for Mental Health Online/ Psychiatric Society for Informatics (Hsiung, 2011)

NBCC – National Board for Certified Counselors (NBCC, 2001)

NZPB – New Zealand Psychologists Board (NZPB, 2011)

Dever (Dever Fitzgerald, Hunter, Hadjistavropoulos, & Koocher, 2010)

Drum (Drum & Littleton, 2014)

Johnson (Johnson, 2014)

Lawlor-Savage (Lawlor-Savage & Prentice, 2014)

Luxton (Luxton, O'Brien, McCann & Mishkind, 2012)
