# Supplementary material for: Consensus Among International Ethical Guidelines for the Provision of Videoconferencing-Based Mental Health Treatments
Source: JMIR Ment Health. 2016 May 18;3(2):e17. doi: 10.2196/mental.5481 (PMC4889868; doi:10.2196/mental.5481)
Supplement: Multimedia Appendix 3 [file mental_v3i2e17_app3.pdf]

	Professional bodies														Published recommendations				
**Recommendation**	**ACA**	**ACPRO**	**AMHCA**	**APA**	**APA D.29**	**APS**	**ATA**	**ATA -SA**	**BPS**	**CPA**	**EFPA**	**ISMHO & PSI**	**NBCC**	**NZPB**	**Dever**	**Drum**	**Johnson**	**Lawlor -Savage**	**Luxton**
General professional competence																			
**Firm recommendations**																			
Psychologists should provide online services within the boundaries of their competence derived from their training, education, supervised experience, or other professional experiences, and should understand the limits and applications of different technologies			X	X		X	X	X			X	X		X	X	X	X		
Psychologists should assist clients to evaluate their competence by verifying their identity for the client (e.g., specifying a geographic location) and making available evidence of their identity, including qualifications, relevant experience (including experience in providing online services) and membership in any official registers/licensing boards and any relevant statutory bodies, and guide the client as to how/where they may verify this information; psychologists' websites should also convey this information in a professional, grammatically-correct and jargon-free manner	X					X	X		X	X	X	X	X	X	X	X			
Psychologists should assume responsibility for continuously evaluating their competencies in this area, including seeking ongoing training	X			X		X	X				X	X		X	X	X	X		
**Tentative recommendations**																			
It may also be reasonable for psychologists to provide prospective clients with guidance as to how to check whether a particular psychologist has had any professional complaints previously filed against them									X										
Psychologists may consider clarifying with clients if an online service will be provided by more than one psychologist (and in this case, to provide the client with a means of verifying each of their identities, professional backgrounds, and affiliations)											X								
It may be reasonable to recommend that any practice or clinic providing e-mental health services should consider providing appropriate training to psychologists in the ethical aspects of e-mental health services																			
It may be reasonable to recommend that psychologists develop adequate skills in 'telepresence' – the ability to use their verbal and nonverbal communications online to engage with the client in a warm, empathic way, develop a collaborative and effective working alliance, and present themselves in a professional, credible manner																	X		
It may be reasonable to recommend that psychologists develop skills to adequately tailor interventions appropriate to individual clients, including cognitive-behavioural therapy, and adapt these appropriately to telepsychological domain, with a good understanding of their applications and limitations																	X		
Technical competence																			
**Firm recommendations**																			

	Professional bodies														Published recommendations				
**Recommendation**	**ACA**	**ACPRO**	**AMHCA**	**APA**	**APA D.29**	**APS**	**ATA**	**ATA -SA**	**BPS**	**CPA**	**EFPA**	**ISMHO & PSI**	**NBCC**	**NZPB**	**Dever**	**Drum**	**Johnson**	**Lawlor -Savage**	**Luxton**
Acquire the requisite skills to manage the practical, technological aspects of the telepsychology methods being used, as well as the 'subtle interpersonal aspects' involved in providing psychological therapy remotely (e.g., developing skills to foster a good working alliance in the absence of visual cues, and skills to deal with communication breakdown and to repair working alliance should technology fail during a session; this should include professional consultation/supervision with experts in e-mental health and undertaking continuing professional development courses targeted towards such skills	X	X			X	X	X							X	X	X	X		X
**Tentative recommendations**																			
Consider monitoring and evaluating the effectiveness and acceptability of the online modality in an ongoing capacity	X											X		X			X		X
Psychologists may consider spending time guiding their clients in mastering practical elements of the technology, in order to support them having a professional, comfortable, and private therapy experience							X												
Psychologists may consider using both structured and freeform methods to regularly elicit feedback regarding the acceptability of online methods									X										
Psychologists may consider scheduling intermittent in-person sessions with a client, to allow for monitoring and evaluation of the online therapy, as well as integrating ongoing monitoring and explicit discussion around the effectiveness of the medium, how technical problems can be managed, and the client's willingness to continue or discontinue according to the functionality of the online modality					X													X	
In assessing suitability of service																			
**Tentative recommendations**																			
Psychologists may consider demonstrating an understanding of the evidence of the effectiveness of the technology-based treatment/intervention, as well as any identified risks the client/s may experience from the delivery of this intervention online, and keep themselves up-to-date with the existing literature as often as it comes out						X		X	X	X				X			X		
Psychologists may consider adequately assessing all prospective clients' needs and presenting problems, and if their apparent needs require an in-person assessment, arrange for an alternative/proxy service to provide this prior to commencing online services										X				X	X		X		
Psychologists may consider refraining from offering online services for which they have not demonstrated their competence to provide the same service in-person										X				X					X
Psychologists may consider regularly monitor clients' progress for therapeutic gains, as well as new situations positing a risk to the client for which management in the online context is not possible or appropriate						X	X							X					X
Psychologists may consider referral to additional and/or alternative FTF services, or more intensive services, when a client's needs or risk profile change so as to require this						X				X							X		

	Professional bodies														Published recommendations				
**Recommendation**	**ACA**	**ACPRO**	**AMHCA**	**APA**	**APA D.29**	**APS**	**ATA**	**ATA -SA**	**BPS**	**CPA**	**EFPA**	**ISMHO & PSI**	**NBCC**	**NZPB**	**Dever**	**Drum**	**Johnson**	**Lawlor -Savage**	**Luxton**
As with FTF services, psychologists may consider being culturally competent to deliver online services to different populations, ensuring that different minority groups are able to equally access and benefit from the online services. This includes an awareness of the client's ethnic or racial background, language, age, gender, sexual orientation, geographic location, socioeconomic and cultural background, providing access to translation if needed	X						X	X	X				X	X	X		X		
Psychologists' may consider making their judgments as to the safety of the online therapy based on research, best practice guidance and client preferences						X													
Given that many prospective clients may be candidates for online therapy precisely due to their remote geographic locations or other barriers to accessing FTF services, psychologists may consider whether the prospective client's has access to any other potential alternative sources of therapy if online therapy is not offered in deciding whether to offer online services														X					
It may be reasonable to recommend that psychologists should have the best interests of clients in mind when determining the appropriateness of offering online services, in order to avoid the 'possible convenience and financial advantages' of online services unduly influencing their treatment recommendations										X									

Note. The abbreviations in the table refer to the following articles. Please see article for full reference list.

ACA – American Counseling Association (ACA, 2014)

ACPRO – Association of Canadian Psychology Regulatory Organizations (ACPRO, 2011)

AMHCA – American Mental Health Counselors Association (AMHCA, 2010)

APA – American Psychological Association (APA, 2013)

APA D.29 - American Psychological Association Division 29 (APA, 2011)

APS – Australian Psychological Society (APS, 2011)

ATA – American Telemedicine Association (ATA, 2013)

ATA-SA – American Telemedicine Association – South Africa (Chipps, Ramlall & Mars, 2012)

BPS – The British Psychological Society (BPS, 2009)

CPA – Canadian Psychological Association (CPA, 2006)

EFPA – European Federation of Psychologists' Association (EFPA, 2006)

ISMHO/PSI – International Society for Mental Health Online/ Psychiatric Society for Informatics (Hsiung, 2011)

NBCC – National Board for Certified Counselors (NBCC, 2001)

NZPB – New Zealand Psychologists Board (NZPB, 2011)

Dever (Dever Fitzgerald, Hunter, Hadjistavropoulos, & Koocher, 2010)

Drum (Drum & Littleton, 2014)

Johnson (Johnson, 2014)

Lawlor-Savage (Lawlor-Savage & Prentice, 2014)

Luxton (Luxton, O'Brien, McCann & Mishkind, 2012)
