# Supplementary material for: Consensus Among International Ethical Guidelines for the Provision of Videoconferencing-Based Mental Health Treatments
Source: JMIR Ment Health. 2016 May 18;3(2):e17. doi: 10.2196/mental.5481 (PMC4889868; doi:10.2196/mental.5481)
Supplement: Multimedia Appendix 4 [file mental_v3i2e17_app4.pdf]

	Professional bodies														Published recommendations				
**Recommendation**	**ACA**	**ACPRO**	**AMHCA**	**APA**	**APA D.29**	**APS**	**ATA**	**ATA -SA**	**BPS**	**CPA**	**EFPA**	**ISMHC & PSI**	**NBCC**	**NZPB**	**Dever**	**Drum**	**Johnson**	**Lawlor -Savage**	**Luxton**
Psychologist registration/accreditation																			
**Firm recommendations**																			
Psychologists should know and comply with all relevant laws and regulations (both for the location where they are practising from, and for where the client is located) when providing online therapeutic services to clients across jurisdictional and/or international borders, including determining whether online psychological therapy is permitted in that jurisdiction, or whether any restrictions apply	X	X	X	X	X	X	X	X	X	X		X	X	X	X				X
Psychologists should ensure that their relevant licensing board approves the provision of online services and to check whether they need to undertake site-specific credentialing procedures to acquire valid registrations/practice licenses across two jurisdictions	X	X	X		X	X		X		X		X	X	X			X		X
**Tentative recommendations**																			
Psychologists may consider that legislative differences across jurisdictions may impact not only whether online services are permitted, but also other relevant regulatory/professional obligations such as the informed consent processes, and mandatory reporting of child abuse or other persons at risk to themselves/others	X	X	X							X			X	X					X
Psychologists may consider ensuring that their malpractice/professional indemnity insurance also covers them for the provision of online services		X			X	X		X		X			X	X			X		
Psychologists may consider discussing relevant ethical regulations and limitations related to the client’s and psychologist’s jurisdiction	X																X		
If the laws/regulations conflict between the psychologist’s jurisdiction and that of their client, psychologists may consider adhering to the higher, or more conservative, standard		X					X												
In cases where there are no specific laws or regulations in place relating to the provision of e-mental health, it may be reasonable that psychologists provide a ‘usual, ethical’ standard of care					X														
Record keeping and electronic records																			
**Tentative recommendations**																			
Psychologists may consider adhering to the usual laws and professional standards regarding thorough record keeping, particularly in cases where their practise diverges from usual practice in case of an adverse event	X				X	X	X	X			X	X		X	X				
Psychologists may consider ensuring the security and back up of any information stored electronically, as long as legally required						X	X		X		X			X					
Psychologists may consider storing electronic materials and other records in a manner that ensures ongoing accessibility to these electronic records as technology advances									X					X					

	Professional bodies														Published recommendations				
**Recommendation**	**ACA**	**ACPRO**	**AMHCA**	**APA**	**APA D.29**	**APS**	**ATA**	**ATA -SA**	**BPS**	**CPA**	**EFPA**	**ISMHO & PSI**	**NBCC**	**NZPB**	**Dever**	**Drum**	**Johnson**	**Lawlor -Savage**	**Luxton**
Psychologists may consider keeping records of all electronic communications with clients (eg., emails) in order to have an accurate account of psychologist-client interactions/conversations						X		X	X										
Psychologists may be advised to record and store audio-visual data from the online psychological sessions themselves, being aware of its subsequent potential use in supervision, research, and legal proceedings													X	X					
If psychologists keep audio-visual recordings from the online therapeutic sessions themselves, they may consider informing clients that these recordings are being kept											X	X	X	X					
It may be reasonable to recommend that any audio-visual recordings (or other electronic material) should be stored confidentially, and not released these without specific client authorization							X					X	X		X				
Psychologists should consider clearly inform clients about their rights regarding access to and deletion of their personal data stored on any electronic media	X								X		X		X		X				
It may be reasonable to recommend that psychologists clearly document their rationale for choosing a particular therapy/modality with a particular client																	X		
It may be reasonable for psychologists to be aware that the same professional standards could be applied to email/other written online interactions in the case of a complaint														X					
It may be reasonable for electronic material (e.g., email) to be stored in hard copies										X									
It may be reasonable for psychologists to maintain a comprehensive electronic record for each client for whom they provide online services, including an assessment, identification and contact information, history, treatment plan, informed consent, information about fees/billing, a documented treatment plan, and documented records for all forms of communication with the client (eg., written, audio-visual, verbal)							X												
It may be reasonable to recommend that psychologists be aware that online methods created the potential for clients to record/store information relating to the online interaction											X								
Billing																			
**Tentative recommendations**																			
Psychologists may consider arrange secure payment methods, and clarify with prospective clients up-front the structure and nature of charges for each session, and whether this differs according to online service type (eg., group versus individual session)						X			X					X	X				
Given the potential for complexities in this area, it may be reasonable to recommend that psychologists closely consult their insurance carriers to ensure proper provision for reimbursement prior to commencing services with a prospective client					X														

	Professional bodies														Published recommendations				
**Recommendation**	**ACA**	**ACPRO**	**AMHCA**	**APA**	**APA D.29**	**APS**	**ATA**	**ATA -SA**	**BPS**	**CPA**	**EFPA**	**ISMHO & PSI**	**NBCC**	**NZPB**	**Dever**	**Drum**	**Johnson**	**Lawlor -Savage**	**Luxton**
It may be reasonable to recommend that psychologists clearly stipulate on invoices/billing documentation that the service had been provided via electronic means when billing to a third party for the purposes of transparency (to avoid fraud)														X					
Establishing age/capacity for consent																			
**Tentative recommendations**																			
Psychologists may consider taking steps to establish the age and maturity of a prospective client who is a young person, to determine the appropriateness of offering that online service						X							X	X	X				
Psychologists may consider obtaining and verifying the consent of a parent/guardian for a minor before services can proceed						X					X		X		X				
It may be reasonable to recommend that in providing online services to a young person, a psychologist should assume responsibility for both the young person and to the 'client-parent' who contracts or engages the psychological service for the young person						X													
Psychologists may consider discussing and clarifying issues of consent, confidentiality and disclosure prior to the commencement of online services						X													

Note. The abbreviations in the table refer to the following articles. Please see article for full reference list.
ACA – American Counseling Association (ACA, 2014)
ACPRO – Association of Canadian Psychology Regulatory Organizations (ACPRO, 2011)
AMHCA – American Mental Health Counselors Association (AMHCA, 2010)
APA – American Psychological Association (APA, 2013)
APA D.29 - American Psychological Association Division 29 (APA, 2011)
APS – Australian Psychological Society (APS, 2011)
ATA – American Telemedicine Association (ATA, 2013)
ATA-SA – American Telemedicine Association – South Africa (Chipps, Ramlall & Mars, 2012)
BPS – The British Psychological Society (BPS, 2009)
CPA – Canadian Psychological Association (CPA, 2006)
EFPA – European Federation of Psychologists' Association (EFPA, 2006)
ISMHO/PSI – International Society for Mental Health Online/ Psychiatric Society for Informatics (Hsiung, 2011)
NBCC – National Board for Certified Counselors (NBCC, 2001)
NZPB – New Zealand Psychologists Board (NZPB, 2011)
Dever (Dever Fitzgerald, Hunter, Hadjistavropoulos, & Koocher, 2010)
Drum (Drum & Littleton, 2014)
Johnson (Johnson, 2014)
Lawlor-Savage (Lawlor-Savage & Prentice, 2014)
Luxton (Luxton, O'Brien, McCann & Mishkind, 2012)
