# Supplementary material for: Consensus Among International Ethical Guidelines for the Provision of Videoconferencing-Based Mental Health Treatments
Source: JMIR Ment Health. 2016 May 18;3(2):e17. doi: 10.2196/mental.5481 (PMC4889868; doi:10.2196/mental.5481)
Supplement: Multimedia Appendix 5 [file mental_v3i2e17_app5.pdf]

	Professional bodies														Published recommendations				
**Recommendation**	**ACA**	**ACPRO**	**AMHCA**	**APA**	**APA D.29**	**APS**	**ATA**	**ATA -SA**	**BPS**	**CPA**	**EFPA**	**ISMHC & PSI**	**NBCC**	**NZPB**	**Dever**	**Drum**	**Johnson**	**Lawlor -Savage**	**Luxton**
Privacy during session																			
**Firm recommendations**																			
Psychologists should take all reasonable, up-to-date precautionary efforts (e.g., computer security measures) to protect and maintain the confidentiality of data/information relating to their clients, in addition to informing them of all precautions taken, and any increased risks to confidentiality inherent to different technologies used (e.g., email versus videoconferencing) as well as limits to confidentiality (eg., in the case of a subpoena)	X			X		X		X	X	X	X	X	X	X	X				X
**Tentative recommendations**																			
Psychologists may consider providing prospective clients with a plainly-written, easy-to-understand ‘terms and conditions’ type statement regarding the service							X		X						X				
Psychologists may consider avoiding unencrypted online platforms (e.g., Skype), particularly for any storage of personal information, to maintain privacy	X				X	X			X				X		X				X
Psychologists may consider discussing security risks with clients through documented informed consent conversations	X				X	X				X		X	X		X			X	
It may be reasonable to recommend that psychologists discuss with clients what software/hardware they may need to participate in the online service securely, and if the psychologist is able to assist in the provision of any of this equipment										X									
It may be reasonable to recommend that psychologists avoid conducting videoconferencing-based sessions while they are in public environments (e.g., café, library)																X			
It may be reasonable to recommend that psychologists advise their clients to ensure a private, confidential environment in which online sessions may take place; this may involve discussing the client identifying a private room, with a clocked door to prevent interruptions																X			
Psychologists may also consider advising clients to take measures to protect the identity of others who may be in their environments, for example by ensuring that family, friends, or roommates do not enter the room, or by keeping family photographs out of the view of the web-camera																X			
Client anonymity and establishing identity																			
**Tentative recommendations**																			
Psychologists may consider generally requiring the user to be identified/identifiable as part of ‘usual practice’	X		X				X	X		X				X					
Psychologists may consider taking steps to verify the client’s identity in cases where this is necessary, for example by scheduling an initial in-person or video-conferencing meeting prior to other services, sighting a government-issued photo ID, a credit card transaction, using code words/numbers, or validating an email address, but to be aware that even these methods of identity-verification may still be open to misuse	X	X	X			X	X	X	X	X			X						

	Professional bodies														Published recommendations				
**Recommendation**	**ACA**	**ACPRO**	**AMHCA**	**APA**	**APA D.29**	**APS**	**ATA**	**ATA -SA**	**BPS**	**CPA**	**EFPA**	**ISMHC & PSI**	**NBCC**	**NZPB**	**Dever**	**Drum**	**Johnson**	**Lawlor -Savage**	**Luxton**
Psychologists may consider that a prospective client's request to remain anonymous may or may not be practical or ethical depending on the circumstances, and consider the nature and risks associated with the service in determining the appropriateness of a request to remain anonymous									X		X								
In cases where the client remains anonymous, consider addressing with clients, limitations to this service, or circumstances under which this arrangement would no longer be tenable (e.g., changes in level of risk) as well as how this situation would be managed (e.g., agreed-upon risk management strategies)									X		X								
It may be reasonable to recommend that psychologists remain vigilant to apparent discrepancies in the client's presentation (e.g., relating to their identity) and clarify the source and nature of information presented as much as possible						X		X											
It may be reasonable to recommend that psychologists be aware that online services enable clients to remain anonymous (even surreptitiously, e.g., providing a false identity), and consequently the information a client may disclose may be misleading or false						X		X											
It may be reasonable to recommend that psychologists also document the location where the client would be receiving services							X												
Ensuring confidentiality in the use/storage of electronic material																			
**Tentative recommendations**																			
Psychologists may consider providing clients with a statement indicating what and how information would be collected, used, disclosed, and stored						X						X	X						
Psychologists may consider taking reasonable efforts to dispose of data/information in a manner that maintains this confidentiality and continues to protect the data against unauthorized access			X	X													X		
Psychologists may consider not forwarding any electronic copies of communications with a client (e.g., email, SMS, or other audio-visual recording) to others without the client's specific consent			X			X							X						
Psychologists may also consider agreeing with clients on a mechanism by which clients could confirm that they had received an email from the psychologist (eg., auto-reply including the psychologist's original message)						X													

Note. The abbreviations in the table refer to the following articles. Please see article for full reference list.

ACA – American Counseling Association (ACA, 2014)

ACPRO – Association of Canadian Psychology Regulatory Organizations (ACPRO, 2011)

AMHCA – American Mental Health Counselors Association (AMHCA, 2010)

APA – American Psychological Association (APA, 2013)

APA D.29 - American Psychological Association Division 29 (APA, 2011)

APS – Australian Psychological Society (APS, 2011)

ATA – American Telemedicine Association (ATA, 2013)
ATA-SA – American Telemedicine Association – South Africa (Chipps, Ramlall & Mars, 2012)
BPS – The British Psychological Society (BPS, 2009)
CPA – Canadian Psychological Association (CPA, 2006)
EFPA – European Federation of Psychologists' Association (EFPA, 2006)
ISMHO/PSI – International Society for Mental Health Online/ Psychiatric Society for Informatics (Hsiung, 2011)
NBCC – National Board for Certified Counselors (NBCC, 2001)
NZPB – New Zealand Psychologists Board (NZPB, 2011)
Dever (Dever Fitzgerald, Hunter, Hadjistavropoulos, & Koocher, 2010)
Drum (Drum & Littleton, 2014)
Johnson (Johnson, 2014)
Lawlor-Savage (Lawlor-Savage & Prentice, 2014)
Luxton (Luxton, O'Brien, McCann & Mishkind, 2012)
