# Supplementary material for: Consensus Among International Ethical Guidelines for the Provision of Videoconferencing-Based Mental Health Treatments
Source: JMIR Ment Health. 2016 May 18;3(2):e17. doi: 10.2196/mental.5481 (PMC4889868; doi:10.2196/mental.5481)
Supplement: Multimedia Appendix 6 [file mental_v3i2e17_app6.pdf]

	Professional bodies														Published recommendations				
**Recommendation**	**ACA**	**ACPRO**	**AMHCA**	**APA**	**APA D.29**	**APS**	**ATA**	**ATA -SA**	**BPS**	**CPA**	**EFPA**	**ISMHC & PSI**	**NBCC**	**NZPB**	**Dever**	**Drum**	**Johnson**	**Lawlor -Savage**	**Luxton**
Confidentiality limits and the content of consent for e-mental health																			
**Firm recommendations**																			
Psychologists should obtain and document thorough informed consent whenever possible, compliant with all relevant laws and regulations.	X			X	X	X	X			X	X	X		X			X	X	
Consent for e-mental health services should address key issues relevant to the technology as well as the process of therapy, including: (limits to) privacy and confidentiality, the structure and timing of services provided, potential risks, limitations unique to the media being used and to what service will/can be provided online, fees, security steps taken, reliability of the online connection, technological equipment and skills requirements, limits to communication and the possibility for misunderstandings to occur, record keeping (what and how personal information will be recorded and stored and who will have access to it), risk management strategies, mandatory reporting, contact availability times, conditions of participation/termination, and cancellation policies, as well as the available of alternatives to receiving psychological treatment online	X		X	X	X	X	X	X		X		X		X	X			X	
**Tentative recommendations**																			
Psychologists may consider providing clients with a hard-copy ‘information sheet’ and hard copy signed consent contracts, to formalise the therapeutic relationship														X					
Psychologists may consider also appropriately informing clients as to the potential benefits to them in engaging in psychological services on the internet	X					X				X		X		X					
Psychologists may consider appropriately informing clients as to the evidence-based nature of the service, or the lack of such evidence									X					X					
Psychologists may consider explicitly addressing and providing technical information needed to access and navigate the online platforms being used; this may include an initial session to test the technology and/or a home visit to assist the client to set-up the equipment prior to the first clinical encounter	X					X				X			X						X
Psychologists may consider explicitly discussing any expectations or restrictions on clients copying or recording the sessions												X		X		X			
Psychologists may consider explicitly addressing the issue of whether it is appropriate for the client to forward or share any written advice from the psychologist; psychologist should also consider implementing measures that restrict a client’s ability to download any video or audio files to their computers, or copy written text						X										X			
Psychologists may consider informing prospective clients as to how complaints may be lodged (in the client’s jurisdiction) should that need arise		X												X			X		
It may be reasonable to recommend that the process of undertaking informed consent needs to occur in ‘real time’							X												
Clarifying contact times																			

	Professional bodies														Published recommendations				
**Recommendation**	**ACA**	**ACPRO**	**AMHCA**	**APA**	**APA D.29**	**APS**	**ATA**	**ATA -SA**	**BPS**	**CPA**	**EFPA**	**ISMHO & PSI**	**NBCC**	**NZPB**	**Dever**	**Drum**	**Johnson**	**Lawlor -Savage**	**Luxton**
**Tentative recommendations**																			
It may be reasonable to recommend that psychologists clarify contact information for both psychologist and client, as well as expectations regarding between-session contact availability (in what form, when, and under what conditions, if any) at the commencement of treatment	X					X	X		X	X		X	X	X	X				
Psychologists may consider specifying specific time frames for expected responses between sessions (e.g., expected time to respond to an email or check a voicemail message), and include in this a discussion around alternative emergency contacts and risk management, as well as processes for contacting the psychologist, should electronic forms of communication breakdown	X					X	X		X			X	X	X	X	X			
Psychologists may consider the importance of ‘setting appropriate boundaries’ around contact times	X									X									
Capacity to provide consent																			
**Tentative recommendations**																			
It may be reasonable to recommend that psychologists be wary to verify that their prospective client is capable of providing informed consent, given that minors may also be highly computer literate									X	X		X	X	X					
Psychologists may consider using an in-person contracting session to enable informed consent (or informed assent with a parent/guardian’s consent) to enable the identity of the substitute decision maker/consenter to be verified in person either with the psychologist or another qualified health practitioner										X		X		X	X				
In some cases where a parent/guardian’s consent is not possible to obtain, it may be reasonable that psychologists continue to offer online services and collaborate with real-world resources and support services might be engaged to supplement the online therapy														X					

Note. The abbreviations in the table refer to the following articles. Please see article for full reference list.
ACA – American Counseling Association (ACA, 2014)
ACPRO – Association of Canadian Psychology Regulatory Organizations (ACPRO, 2011)
AMHCA – American Mental Health Counselors Association (AMHCA, 2010)
APA – American Psychological Association (APA, 2013)
APA D.29 - American Psychological Association Division 29 (APA, 2011)
APS – Australian Psychological Society (APS, 2011)
ATA – American Telemedicine Association (ATA, 2013)
ATA-SA – American Telemedicine Association – South Africa (Chipps, Ramlall & Mars, 2012)
BPS – The British Psychological Society (BPS, 2009)
CPA – Canadian Psychological Association (CPA, 2006)
EFPA – European Federation of Psychologists’ Association (EFPA, 2006)
ISMHO/PSI – International Society for Mental Health Online/ Psychiatric Society for Informatics (Hsiung, 2011)
NBCC – National Board for Certified Counselors (NBCC, 2001)
NZPB – New Zealand Psychologists Board (NZPB, 2011)

Dever (Dever Fitzgerald, Hunter, Hadjistavropoulos, & Koocher, 2010)
Drum (Drum & Littleton, 2014)
Johnson (Johnson, 2014)
Lawlor-Savage (Lawlor-Savage & Prentice, 2014)
Luxton (Luxton, O'Brien, McCann & Mishkind, 2012)
