# Supplementary material for: Consensus Among International Ethical Guidelines for the Provision of Videoconferencing-Based Mental Health Treatments
Source: JMIR Ment Health. 2016 May 18;3(2):e17. doi: 10.2196/mental.5481 (PMC4889868; doi:10.2196/mental.5481)
Supplement: Multimedia Appendix 7 [file mental_v3i2e17_app7.pdf]

	Professional bodies														Published recommendations				
**Recommendation**	**ACA**	**ACPRO**	**AMHCA**	**APA**	**APA D.29**	**APS**	**ATA**	**ATA -SA**	**BPS**	**CPA**	**EFPA**	**ISMHC & PSI**	**NBCC**	**NZPB**	**Dever**	**Drum**	**Johnson**	**Lawlor -Savage**	**Luxton**
Preventing 'boundary crossings'																			
**Tentative recommendations**																			
It may be reasonable to recommend that psychologists consider the greater potential for boundary as well as privacy issues to emerge using online media						X						X		X			X		
Psychologists may consider maintaining the same level of professional language across all media as they would in person, including avoiding the use of abbreviations or text-message shorthand, and 'emoticons', even when the client uses these, in order to avoid acquiring a 'dual role' (e.g., as 'friend'); psychologists should also review their written feedback prior to sending it to clients to check for alternative interpretations, engage in regular paraphrasing of their intended message, and check the client's understanding of the message						X								X		X		X	
Psychologists may consider clarifying with clients the extent of electronic communication to be expected (e.g., email, text messages) and the speed or 'turnaround time' that clients can expect before they receive a response; up to 24 hours may be acceptable for more instant communications, while 1-3 business days is generally considered acceptable for feedback regarding progress on an online program/home practice	X					X										X			
Psychologists may consider avoiding quick, 'rapid fire' responding to emails they receive from clients, to avoid clients becoming overly dependent upon the psychologist being readily available; if psychologists perceive that their client's speed of communication is excessive and clinically contra-indicated, the psychologist should model a more appropriate therapeutic interaction pace by slowing their own responses down.														X		X			
Psychologists using videoconferencing-based services may consider taking steps to create a safe and professional space, by having a consistent, professional-looking background to their video, that is sensitive to the cultural background of their client, and by dressing in a professional manner -								X								X			
It may be reasonable to recommend that psychologists explicitly address any boundary crossings/issues related to email or other electronic communication be in-session														X					
It may be reasonable to recommend that psychologists only schedule and undertake online e-mental health services during normal business hours, and respect the timing of appointments in the same manner as FTF transactions																X			
It may be reasonable to recommend that psychologists model appropriate boundaries and self-care by not continuing to schedule online services when ill, on vacation, weekends or other personal/family time; this may include notifying clients of scheduled leave time in advance, clarifying when therapeutic communications may recommence																X			

	Professional bodies														Published recommendations				
**Recommendation**	**ACA**	**ACPRO**	**AMHCA**	**APA**	**APA D.29**	**APS**	**ATA**	**ATA -SA**	**BPS**	**CPA**	**EFPA**	**ISMHO & PSI**	**NBCC**	**NZPB**	**Dever**	**Drum**	**Johnson**	**Lawlor -Savage**	**Luxton**
With the exception of clients in crisis, it may be reasonable to recommend that psychologists provide clients with responses to between-session communications in the order that they are received, in order to prevent unintentional favoritism																X			
Psychologists may consider using strategies to prevent excessive background noise whilst videoconferencing, for example by using a white-noise machine outside of their office door, or by delivering the session from a soundproof room																X			
Social media																			
**Tentative recommendations**																			
It may be reasonable to recommend that psychologists take as many reasonable steps as possible to protect their own privacy, for example by not accepting 'friend requests' on Facebook and explicitly addressing the need for this boundary with the client; this may include having a standard policy in place regarding how nontherapeutic online contacts will be handled, and discussing this policy up-front with clients	X					X										X			
Psychologists may also consider that any personal information posted on personal social networking sites or other online domains/blogs/communities may be widely accessible and even with privacy settings, should be considered "public" ; therefore separate professional and personal social media presence may be recommended	X					X										X			
Psychologists may consider only searching for further information about a client (e.g., Google search) if it is in the best interests of the client, for example in a situation where other people may be at risk, and/or in a situation where consent has been given, and not simply to satisfy the client's curiosity	X					X													
It may be reasonable to recommend that psychologists monitor personal information available about them on the internet (e.g., social media, blogs, dating websites, and even records of professional behaviour or extra-curricular activities) and take steps to remove any inappropriate content						X													
It may be reasonable to recommend that psychologists consider the impact of a client learning any of this information (e.g., from a psychologist's blog) on the therapeutic relationship, and place the client's best interests first and foremost						X													
If the psychologist does undertake searches about a client, it may be reasonable to recommend that psychologists declare the source of any information they obtain that is the basis for subsequent decision making (e.g., suicidal inclinations, dysfunctional dating behaviour) and give the client an opportunity to address the psychologist's concerns, as acting on information the person has not provided themselves may be 'inconsistent with a respect for the individual's privacy and autonomy'														X					

Note. The abbreviations in the table refer to the following articles. Please see article for full reference list.
ACA – American Counseling Association (ACA, 2014)
ACPRO – Association of Canadian Psychology Regulatory Organizations (ACPRO, 2011)
AMHCA – American Mental Health Counselors Association (AMHCA, 2010)
APA – American Psychological Association (APA, 2013)
APA D.29 - American Psychological Association Division 29 (APA, 2011)
APS – Australian Psychological Society (APS, 2011)
ATA – American Telemedicine Association (ATA, 2013)
ATA-SA – American Telemedicine Association – South Africa (Chipps, Ramlall & Mars, 2012)
BPS – The British Psychological Society (BPS, 2009)
CPA – Canadian Psychological Association (CPA, 2006)
EFPA – European Federation of Psychologists' Association (EFPA, 2006)
ISMHO/PSI – International Society for Mental Health Online/ Psychiatric Society for Informatics (Hsiung, 2011)
NBCC – National Board for Certified Counselors (NBCC, 2001)
NZPB – New Zealand Psychologists Board (NZPB, 2011)
Dever (Dever Fitzgerald, Hunter, Hadjistavropoulos, & Koocher, 2010)
Drum (Drum & Littleton, 2014)
Johnson (Johnson, 2014)
Lawlor-Savage (Lawlor-Savage & Prentice, 2014)
Luxton (Luxton, O'Brien, McCann & Mishkind, 2012)
