# Supplementary material for: Consensus Among International Ethical Guidelines for the Provision of Videoconferencing-Based Mental Health Treatments
Source: JMIR Ment Health. 2016 May 18;3(2):e17. doi: 10.2196/mental.5481 (PMC4889868; doi:10.2196/mental.5481)
Supplement: Multimedia Appendix 8 [file mental_v3i2e17_app8.pdf]

	Professional bodies														Published recommendations				
**Recommendation**	**ACA**	**ACPRO**	**AMHCA**	**APA**	**APA D.29**	**APS**	**ATA**	**ATA -SA**	**BPS**	**CPA**	**EFPA**	**ISMHO & PSI**	**NBCC**	**NZPB**	**Dever**	**Drum**	**Johnson**	**Lawlor -Savage**	**Luxton**
Crisis management strategies																			
**Firm recommendations**																			
Psychologists should establish in-person clinical supports in the client's geographic location in case of emergency, such as a primary care provider, prior to initiating online treatment					X	X	X	X		X		X	X	X	X				X
**Tentative recommendations**																			
It may be reasonable to recommend that psychologists continue to monitor all clients throughout online therapy, including those who do not initially present as 'high risk', as their level of risk may change or increase over the course of the online treatment					X									X			X		X
Psychologists may consider knowing the phone numbers for the local police department, emergency response teams, or crisis mental health teams in the client's jurisdiction, and have these telephone numbers on hand at the start of each online session					X		X			X		X	X	X					X
Where possible and relevant, it may be recommended that psychologists obtain and keep the contact details of the client's next of kin or other preferred (personal) emergency contact						X	X			X				X	X				X
In a situation where risk emerges, and the psychologist knows the clients identity, it may be recommended that they stay engaged with the client, obtain as many identifying details as they can, in order that the risk of harm may be reduced and/or the client can be referred to other appropriate services. In addition, it was recommended that the psychologist consults with another suitably informed colleague(s) to assist in their decision-making						X													X
Where psychologists do know the client's identity, tentative recommendations suggest that psychologists are bound by the same reporting obligations should they become aware that their client (or someone else) is at risk						X													
It may be reasonable to recommend that psychologists' consideration of crisis management depends upon whether the client will be participating in online services alone or in the company of another health care professional. If the client is in a setting with other immediately-available health-care professionals (e.g., a local clinic, school, library, outpatient clinic) it is important that the psychologist becomes familiar with that facility's emergency procedures, or if these do not exist, to coordinate with the facility in devising a basic set of procedures including identifying nearest local emergency resources and phone numbers (and possibly their average response times), becoming familiar with the nearest hospital emergency room able to manage a mental health emergency/psychiatric admission, and having the patient's family or emergency contact information								X											
Communication of crisis management strategies																			
**Tentative recommendations**																			

	Professional bodies														Published recommendations				
**Recommendation**	**ACA**	**ACPRO**	**AMHCA**	**APA**	**APA D.29**	**APS**	**ATA**	**ATA -SA**	**BPS**	**CPA**	**EFPA**	**ISMHO & PSI**	**NBCC**	**NZPB**	**Dever**	**Drum**	**Johnson**	**Lawlor -Savage**	**Luxton**
Psychologists may consider discussing crisis management procedures prior to the commencement of online psychological therapy, through the intake process	X									X		X		X			X	X	X
Psychologists may consider determining local supports and preferred local health-care providers in collaboration with the client										X			X	X			X		X
Psychologists inform clients of alternative means of communication both for them, and for the client, should the technology fail	X	X								X			X	X	X		X		X
Psychologists may consider ensuring adequate methods of checking and responding to messages from the client in times of their unavailability, illness or absence										X				X			X		
Psychologists may consider also addressing the possibility that they may not receive an electronic communication immediately, and address their policies and expectations around this												X					X		
Psychologist may consider discussing with their clients how the client could cope with potential misunderstandings related to the technology (e.g., loss of visual cues); this may include discussing how text-based “chat” can be used to supplement videoconferencing-based communication should the audio and/or visual cues be interrupted	X		X										X	X			X	X	
It may be reasonable to recommend that psychologists’ decision-making regarding whether e-mental health services are appropriate for a particular client is contingent upon the collaborative determination of a ‘crisis support plan’ in case of emergency						X													
It may be reasonable to recommend that psychologists discuss up front any collaboration and communication that would take place with other multidisciplinary members of a treatment team or other involved health-care practitioners, provided the patient is consenting							X												
It may be reasonable to recommend that psychologists discuss with their client in what circumstances the patient and psychologist’s emergency contacts can be used																			
Psychologist’s responsibilities																			
**Tentative recommendations**																			
It may be reasonable to recommend that psychologists be familiar with mandatory reporting and involuntary hospitalisation laws both for their jurisdiction, and the jurisdiction in which their client resides and is receiving services							X	X						X					X
Psychologists may consider having a good understanding of technical issues that may arise, and have appropriate risk management protocols in place (eg., protocols if a distressed patient leaves session abruptly)							X										X		
It may be recommended that that psychologists confirm the patient’s current location and whether there have been any changes to their personal support system at the commencement of each session							X												X

	Professional bodies														Published recommendations				
**Recommendation**	**ACA**	**ACPRO**	**AMHCA**	**APA**	**APA D.29**	**APS**	**ATA**	**ATA -SA**	**BPS**	**CPA**	**EFPA**	**ISMHO & PSI**	**NBCC**	**NZPB**	**Dever**	**Drum**	**Johnson**	**Lawlor -Savage**	**Luxton**
In circumstances where psychologists are providing online psychological services to anonymous clients, it may be reasonable to recommend that psychologists 'offset' the risks involved in this by providing clients with clear, written instructions regarding what to do in a mental health emergency (e.g., suicide risk) and discuss these arrangements in professional supervision meetings														X					
It may be reasonable to recommend that psychologists are aware of any medications their client may be taking, as well as the prescribing health-care professionals for these medications should the client experience any apparent side effects, increase in symptoms, or other medication-related issues							X												
It may be reasonable to recommend that psychologists are prepared and able to act on their emergency/risk management procedures even when the client themselves (or their emergency contact) is uncooperative							X												
As clients engaging with online services may be even more likely to have transportational barriers (e.g., live in a geographically remote area), which may impact their capacity to self-transport or gain access to in-person services in the case of emergency, it may be reasonable to recommend that psychologists have a contingency plan for this and liaise with the client's support person/emergency contact and the services themselves to identify strategies to overcome these limitations							X												

Note. The abbreviations in the table refer to the following articles. Please see article for full reference list.
ACA – American Counseling Association (ACA, 2014)
ACPRO – Association of Canadian Psychology Regulatory Organizations (ACPRO, 2011)
AMHCA – American Mental Health Counselors Association (AMHCA, 2010)
APA – American Psychological Association (APA, 2013)
APA D.29 - American Psychological Association Division 29 (APA, 2011)
APS – Australian Psychological Society (APS, 2011)
ATA – American Telemedicine Association (ATA, 2013)
ATA-SA – American Telemedicine Association – South Africa (Chipps, Ramlall & Mars, 2012)
BPS – The British Psychological Society (BPS, 2009)
CPA – Canadian Psychological Association (CPA, 2006)
EFPA – European Federation of Psychologists' Association (EFPA, 2006)
ISMHO/PSI – International Society for Mental Health Online/ Psychiatric Society for Informatics (Hsiung, 2011)
NBCC – National Board for Certified Counselors (NBCC, 2001)
NZPB – New Zealand Psychologists Board (NZPB, 2011)
Dever (Dever Fitzgerald, Hunter, Hadjistavropoulos, & Koocher, 2010)
Drum (Drum & Littleton, 2014)
Johnson (Johnson, 2014)
Lawlor-Savage (Lawlor-Savage & Prentice, 2014)
Luxton (Luxton, O'Brien, McCann & Mishkind, 2012)
