# Supplementary material for: Consensus Among International Ethical Guidelines for the Provision of Videoconferencing-Based Mental Health Treatments
Source: JMIR Ment Health. 2016 May 18;3(2):e17. doi: 10.2196/mental.5481 (PMC4889868; doi:10.2196/mental.5481)
Supplement: Multimedia Appendix 9 [file mental_v3i2e17_app9.pdf]

Professional bodies

	ACA	ACPRO	AMHCA	APA-JT	APA D.29	APS	ATA	ATA-SA	BPS	CPA	EFPA	ISMHO & PSI	NBCC	NZPB
**Over-arching ethical obligations do not change when using e-mental health**		X	X	X		X	X	X	X		X		X	X
**Appropriateness of e-mental health**														
Diagnosis/issue	X		X	X				X						X
Personal factors	X		X	X										X
Limits of non-verbal cues	X													X
Cultural factors	X			X				X					X	X
Type of service						X		X						X
**Competence**														
Professional competence	X	X	X	X		X	X	X			X	X		X
Technological	X	X		X	X									X
Assessing suitability of service	X			X		X		X		X	X		X	X
**Registration/ legal issues**														
Registration/ accreditation	X	X	X	X	X	X	X	X	X	X		X	X	X
Record keeping	X				X			X		X	X	X	X	X
Billing				X					X					X
Establishing age/ capacity for consent						X							X	X
**Confidentiality**														
Privacy during session	X			X	X	X	X	X		X		X	X	X
Client anonymity and establishing identity	X	X	X			X		X	X	X	X	X	X	
Electronic storage/security				X		X		X	X	X				X
Email use				X		X				X			X	

Professional bodies

	ACA	ACPRO	AMHCA	APA-JT	APA D.29	APS	ATA	ATA-SA	BPS	CPA	EFPA	ISMHO & PSI	NBCC	NZPB
Videoconferencing						X			X					
**Consent**														
Confidentiality limits	X	X	X	X	X	X		X	X	X		X		X
Clarifying contact times	X				X	X			X	X		X	X	
Capacity					X	X			X	X	X	X		X
**Professional boundaries**														
Preventing crossing boundaries	X											X	X	X
Social media	X			X		X								
**Crisis intervention**														
Strategies to manage crisis	X	X	X	X	X	X		X	X		X	X		X
Communication of strategies	X			X		X			X		X	X	X	X
Psychologists' responsibilities				X		X			X				X	X
**Total domains covered (of 26)**	17	7	8	17	8	18	4	13	12	10	8	11	13	21

Published peer-reviewed recommendations	Dever	Drum	Johnson	Lawlor-Savage	Luxton	Issue coverage across guidelines (of 19)
**Over-arching ethical obligations do not change when using e-mental health**						10
**Appropriateness of e-mental health**						
Diagnosis/issue					X	6
Personal factors				X	X	6
Limits of non-verbal cues			X	X		4
Cultural factors		X			X	7
Type of service			X		X	5
**Competence**						
Professional competence	X	X	X			13
Technological	X	X	X		X	9
Assessing suitability of service	X		X	X	X	12
**Registration/ legal issues**						
Registration/ accreditation	X		X		X	16
Record keeping	X					9
Billing	X					4
**Confidentiality**						
Establishing age/ capacity for consent	X					4
Privacy during session	X	X			X	13
Client anonymity and establishing identity						10
Electronic storage/security	X		X	X	X	10
Email use						4
Videoconferencing				X		3
**Consent**						
Confidentiality limits	X		X	X	X	15
Clarifying contact times		X				8
Capacity	X					8

Published peer-reviewed recommendations	Dever	Drum	Johnson	Lawlor-Savage	Luxton	Issue coverage across guidelines (of 19)
**Professional boundaries**						
Preventing crossing boundaries		X				5
Social media		X				4
**Crisis intervention**						
Strategies to manage crisis	X		X	X	X	15
Communication of strategies	X				X	10
Psychologists' responsibilities	X		X	X	X	9
**Total domains covered (of 26)**	14	7	10	8	13	

Note. The abbreviations in the table refer to the following guidelines: ACA – American Counseling Association (ACA, 2014); ACPRO – Association of Canadian Psychology Regulatory Organizations (ACPRO, 2011); AMHCA – American Mental Health Counselors Association (AMHCA, 2010); APA – American Psychological Association (APA, 2013); APA D.29 - American Psychological Association Division 29 (APA, 2011); APS – Australian Psychological Society (APS, 2011); ATA – American Telemedicine Association (ATA, 2013); ATA-SA – American Telemedicine Association – South Africa (Chipps, Ramlall & Mars, 2012); BPS – The British Psychological Society (BPS, 2009); CPA – Canadian Psychological Association (CPA, 2006); EFPA – European Federation of Psychologists' Association (EFPA, 2006); ISMHO/PSI – International Society for Mental Health Online/ Psychiatric Society for Informatics (Hsiung, 2011); NBCC – National Board for Certified Counselors (NBCC, 2001); NZPB – New Zealand Psychologists Board (NZPB, 2011); Dever (Dever Fitzgerald, Hunter, Hadjistavropoulos, & Koocher, 2010); Drum (Drum & Littleton, 2014); Johnson (Johnson, 2014); Lawlor-Savage (Lawlor-Savage & Prentice, 2014); Luxton (Luxton, O'Brien, McCann & Mishkind, 2012)
