# Supplementary material for: Consensus Among International Ethical Guidelines for the Provision of Videoconferencing-Based Mental Health Treatments
Source: JMIR Ment Health. 2016 May 18;3(2):e17. doi: 10.2196/mental.5481 (PMC4889868; doi:10.2196/mental.5481)
Supplement: Multimedia Appendix 10 [file mental_v3i2e17_app10.pdf]

***Appropriateness of e-mental health***
*Factors related to the client*

- Include an initial process determining appropriateness of online service for client, and refer those not suitable
- Consider relevant research supporting  online provision of service, particularly noting contra-indications
- Consider capacity of patient to arrange practical and technological necessities of the therapy
- Consider their geographic distance to nearest emergency medical facility

*Type of service*

- Be aware that psychological testing/assessments designed for in-person implementation may not be possible/ethical to conduct online
- Clearly communicate inherent risks associated with online and unsupervised test administration to clients

***Competence***
*General professional competence*

- Provide online services within the boundaries of their competence
- Assume responsibility for continuous evaluations of these competencies
- Assists clients in evaluating these competencies

*Technical competence*

- Acquire requisite skills to deliver manage technological aspects of service

*Assessing suitability of service*

- Consider evidence of effectiveness of service, and identify risks of online modality
- Adequately assess needs and presenting problems, consider referral to additional and/or alternative FTF services
- Regularly monitor client's progress
- Remain culturally competent to deliver services to different populations

***Legal and regulatory issues***
*Psychologist registration/accreditation*

- Know and comply with relevant laws and regulations, from both location of their practice and location of client
- Be aware of legislative differences across jurisdictions, and their impact on this service
- Ensure licensing board approves online services, and check relevant credential requirements
- Ensure professional insurance covers online services

*Record keeping and electronic records*

- Adhere to usual laws and professional standards
- Ensure security of any information stored electronically

- Maintain adequate records of electronic communication with clients

*Billing*

- Arrange secure payment methods, and be up-front with charges for services

*Establishing age/capacity for consent*

- Take steps to establish age and maturity of patient, and determine appropriateness of online service
- Obtain and verify consent of parent/guardian where applicable

***Confidentiality***

*Privacy during session*

- Take reasonable efforts to protect and maintain confidentiality of data/information, and inform clients of risks to confidentiality inherent in different technologies
- Discuss security risks with clients in the form of documented consent conversations

*Client anonymity and establishing identity*

- As usual practice, require the user to be identified/identifiable
- Take steps to verify client's identity where necessary

*Ensuring confidentiality in the use/storage of electronic material*

- Provide clients with statement indicating what and how information will be collected, used, disclosed and stored
- Take reasonable efforts to dispose of data/information in a method maintaining confidentiality

***Consent***

*Confidentiality limits and the content of consent for e-mental health*

- Obtain and document thorough informed consent
- Consent should address key issues specific to e-mental health services (e.g. limits to confidentiality, potential risks)
- Inform clients of potential benefits of online services
- Discuss expectations and restrictions of clients recording or copying sessions

*Clarifying contact times*

- Clarify contact information and expectations about between-treatment contact
- Specify time frames for expected response times between sessions, and include emergency contacts

*Capacity to provide consent*

- Be wary, and verify capacity to provide informed consent in young clients
- Use in-person session to enable informed consent

***Professional boundaries***

*Preventing 'boundary crossings'*

- Be aware of greater potential for boundary crossings when using online media
- Maintain same level of professional language across all media as would be appropriate in person

*Social media*

- Take reasonable steps to protect own privacy
- Be aware of posting personal information online
- Only search for further information about a client online when it is in the client's best interest

***Crisis intervention and distress management***

*Crisis management strategies*

- Monitor all clients throughout therapy
- Establish in-person clinical support for client in their geographic location
- Have relevant emergency phone numbers on hand during each session
- Have contact details for client's next of kin where possible
- If risk emerges, remain engaged with client, obtain as many details as possible, and consult with colleagues

*Communication of crisis management strategies*

- Discuss crisis management procedures during intake process
- Collaborate with client to determine local supports for them
- Ensure systems are in place for message checking and response during times of unavailability
- Inform clients of alteration means of communication should technology fail
- Discuss strategies for dealing with potential misunderstandings/miscommunications related to communication via technology

*Psychologists' responsibilities*

- Be familiar with mandatory reporting and involuntary hospitalisation protocols for the jurisdiction of the practice and the client
- Have a good understanding of possible technical issues, and risk management protocols in place for these
- Confirm current location and enquire about any change in personal support system with client at commencement of each session
